# Genome-Wide Characterization and Analysis of *CIPK* Gene Family in Two Cultivated Allopolyploid Cotton Species: Sequence Variation, Association with Seed Oil Content, and the Role of *GhCIPK6*

**DOI:** 10.3390/ijms21030863

**Published:** 2020-01-29

**Authors:** Yupeng Cui, Ying Su, Junjuan Wang, Bing Jia, Man Wu, Wenfeng Pei, Jinfa Zhang, Jiwen Yu

**Affiliations:** 1 State Key Laboratory of Cotton Biology, Institute of Cotton Research of Chinese Academy of Agricultural Sciences, Key Laboratory of Cotton Genetic Improvement, Ministry of Agriculture, Anyang 455000, China; yupeng_cui851026@163.com (Y.C.); Wang20201973@163.com (J.W.); jiabing1814@126.com (B.J.); wuman2004@163.com (M.W.); peiwenfeng1988@163.com (W.P.); 2 Laboratory of Cotton Genetics, Genomics and Breeding, College of Agronomy and Biotechnology/Key Laboratory of Crop Heterosis and Utilization of Ministry of Education/Beijing Key Laboratory of Crop Genetic Improvement, China Agricultural University, Beijing 100193, China; suying0425@163.com; 3 Department of Plant and Environmental Sciences, New Mexico State University, Las Cruces, NM 88003, USA; jinzhang@nmsu.edu

**Keywords:** *Gossypium*, *CIPK*, various stresses, QTLs, expression and regulation, oil content

## Abstract

Calcineurin B-like protein-interacting protein kinases (*CIPKs*), as key regulators, play an important role in plant growth and development and the response to various stresses. In the present study, we identified 80 and 78 *CIPK* genes in the *Gossypium hirsutum* and *G. barbadense*, respectively. The phylogenetic and gene structure analysis divided the cotton *CIPK* genes into five groups which were classified into an exon-rich clade and an exon-poor clade. A synteny analysis showed that segmental duplication contributed to the expansion of *Gossypium CIPK* gene family, and purifying selection played a major role in the evolution of the gene family in cotton. Analyses of expression profiles showed that *GhCIPK* genes had temporal and spatial specificity and could be induced by various abiotic stresses. Fourteen *GhCIPK* genes were found to contain 17 non-synonymous single nucleotide polymorphisms (SNPs) and co-localized with oil or protein content quantitative trait loci (QTLs). Additionally, five SNPs from four *GhCIPKs* were found to be significantly associated with oil content in one of the three field tests. Although most *GhCIPK* genes were not associated with natural variations in cotton oil content, the overexpression of the *GhCIPK6* gene reduced the oil content and increased C18:1 and C18:1+C18:1d6 in transgenic cotton as compared to wild-type plants. In addition, we predicted the potential molecular regulatory mechanisms of the *GhCIPK* genes. In brief, these results enhance our understanding of the roles of *CIPK* genes in oil synthesis and stress responses.

## 1. Introduction

Calcium, as a secondary messenger, plays an important role in plant growth and development and plant responses to environmental stresses [1]. There are four major calcium ion sensors in plants, including calmodulin, calmodulin-like protein, calcium-dependent protein kinase, and calcineurin B-like protein (CBL), which sense and decode the changes of calcium ion concentration in response to various stimuli [2]. Among the four calcium sensors, CBL decodes calcium transients by interacting with and modulating the activity of CBL-interacting protein kinases (CIPKs) in higher plants [3]. CIPKs belong to the sucrose nonfermenting 1 (SNF1) kinase family in plants, which contain three families: SnRK1, SnRK2, and SnRK3, and CIPKs are also known as the SnRK3 family [4,5]. Commonly, CIPK proteins contain a conserved kinase domain in the N-terminus and a regulatory domain in the C-terminus, which is separated by a variable junction domain. The N-terminal domain of CIPKs is similar with the protein structures of SNF1 kinase and AMO-dependent protein kinase. A conserved NAF domain within the C-terminus of CIPKs is required for interacting with CBLs, which activate the catalytic activity of CIPKs, and the activated CIPKs can transfer perceived calcium signals by phosphorylating target downstream signaling components [6,7]. Moreover, a protein phosphatase interaction (PPI) domain in the C-terminus of CIPKs can also interact with specific members of protein phosphatase 2C (PP2C) [8].

Following the identification of 26 *CIPK* family genes in *Arabidopsis thaliana* genome [9], *CIPK* genes in many plant species have also been identified by genome-wide analyses of their genomes. For example, there are 34 in rice (*Oryza sativa*) [10], 43 in maize (*Zea mays*) [11], 27 in poplar (*Populus tremula*) [12], 23 in *Brassica napus* [13], 52 in soybean (*Glycine max*) [14], 20 in *Vitis vinifera* [15], 34 in apple (*Malus domestica*) [16], and 16 *CIPKs* in *Prunus mume* [17]. These studies reported that the *CIPK* gene family can be classified into two groups, including an exon-rich group and exon-poor group. It was reported that *CIPK* genes participate in plant growth and development, and play critical roles in various stresses, including abiotic stresses and hormones [15,18]. The expression levels of *CIPK* genes in *G. raimondii* and *G. arboreum* were induced under abiotic stresses (drought, salt and low temperature) [19]. In *Arabidopsis*, the expression level of *AtCIPK3* was enhanced during the early developmental stages of seedlings, and the *Atcipk3* mutant showed high sensitivity under abscisic acid (ABA) stress during seed germination [20], and overexpression of *GhCIPK6* increased tolerance to salt, drought, and ABA stresses in transgenic *Arabidopsis* [21]. Overexpression of *TaCIPK24* from wheat (*Triticum aestivum* L.) improves salt tolerance and enhances reactive oxygen species (ROS) scavenging capacity in transgenic *Arabidopsis* [18]. The overexpression of *ZmCIPK8* from *Zea mays* confers drought tolerance in transgenic tobacco [22], and overexpression of *TaCIPK27* increases tolerance to drought, but decreases sensitivity to ABA treatment in transgenic *Arabidopsis* [23]. Overexpression of each of the 12 *HsCIPKs* from barley (*Hordeum spontaneum*) in rice shows that all but *HsCIPK28* enhance the tolerance of roots to multiple heavy metal toxicities, and eight *HsCIPKs* (except for *HsCIPK9*, *HsCIPK11*, *HsCIPK14*, and *HsCIPK24*) improve the tolerance to salt, drought, and ABA [24]. Overexpressing the *LlaCIPK* gene from *Lepidium* can enhance cold tolerance in tobacco by increasing the level of proline and cell membrane stability [25]. Although the *CIPK* gene family was reported in *G. arboreum* and *G. raimondii* [19], little is known about their detailed information in *G. hirsutum* and *G. barbadense*, especially their potential roles in various abiotic stresses and cottonseed oil regulation.

Cotton (*Gossypium*) is one of the most important fiber crops around the world, and cottonseed is the main byproduct of cotton, with the oil content of cottonseed ranging from 28.24% to 44.05% and unsaturated fatty acids accounting for the largest component of the oil [26,27]. To explore the genetic composition of oil content is helpful for utilizing cottonseed. Although there are many studies in genetic analysis and quantitative trait locus (QTL) mapping of oil content in different cotton populations [26,28], reports on cotton CIPK proteins are minimal. In *B. napus*, *BnCIPK9* was identified as involved in oil content regulation in F_2_ population; overexpression of *BnCIPK9* in *B. napus* can decrease seed oil content, and seed oil contents of *Atcipk9* mutants were significantly higher than that of wild-type (WT) plants [29]. These results suggest that *CIPK* genes play important roles in carbohydrate and energy metabolism, and oil synthesis. Recently, the whole-genome sequences of four cotton species, including *G. raimondii* [27,30], *G. arboreum* [31,32], *G. hirsutum* acc. TM-1 [33,34,35,36], and *G. barbadense*, acc. 3-79, cv. Xinhai 21 and cv. H7124 [35,36,37,38], provide an opportunity to systematically identify *CIPK* genes in cotton.

In this study, we identified *CIPK* genes from *G. hirsutum* and *G. barbadense*. The identified *CIPKs* were comprehensively analyzed for phylogenetic classifications, gene structures, protein motifs, chromosomal locations, and duplicated genes. Subsequently, gene expression profiles of *GhCIPK* genes were analyzed in different tissues and various abiotic stresses. Finally, the co-localization of *GhCIPK* genes with quantitative trait loci (QTLs) for seed oil or protein content, and sequence variations were analyzed, and the primary function of *GhCIPK6* was also investigated in cottonseed oil synthesis. In addition, alternative splicing events and miRNA target sites of *GhCIPKs* were also predicted. Our study provides a solid foundation for further study of the roles of cotton *CIPK* genes in cotton growth and development, stress responses, and oil synthesis.

## 2. Results

### 2.1. Identification of CIPK Genes in G. hirsutum and G. barbadense

To identify the *CIPK* genes in *G. hirsutum* and *G. barbadense*, we conducted a BLASTP against two cotton genomes with CIPK protein sequences of *Arabidopsis* (26) and rice (34) as queries (Table S1). Then, the InterProscan and SMART databases were used to further verify the putative CIPK proteins. Finally, the two steps resulted in 80 *CIPK* genes from *G. hirsutum* Nanjing Agricultural University (NAU) version, 79 *CIPK* genes from *G. hirsutum* Zhejiang University (ZJU) version, and 79 *CIPK* genes from *G. hirsutum* Huazhong Agricultural University (HAU) version (Table S2). A total of 72, 78, and 77 *CIPK* genes were identified in *G. barbadense* cv. Xinhai 21 genome (NAU version), acc. 3-79 genome (HAU version), and cv. H7124 genome (ZJU version), respectively, using the same methods (Table S3). Information about the protein kinase domain and the NAF domain in each cotton CIPK protein of *G. hirsutum* and *G. barbadense* is listed in Table S4. Combining these results, 80 *GhCIPKs* from *G. hirsutum* NAU version and 78 *GbCIPKs* from *G. barbadense* HAU version were analyzed in the subsequent research.

These cotton *CIPK* genes were named *GhCIPK1*-*GhCIPK80* and *GbCIPK1*-*GbCIPK78* in *G. hirsutum* and *G. barbadense*, respectively, according to their chromosomal positions. The gene name, locus IDs, genomics positions, and other features are shown in Tables S2 and S3. Two genomes of allotetraploid cotton species (*G. hirsutum* and *G. barbadense*) underwent a diploidization following the divergence of *G. arboreum* (A genome) and *G. raimondii* (D genome). Based on the genome scans of two diploid cotton species, the *CIPK* gene family was investigated in *G. raimondii* (41) and *G. arboreum* (39) [19]. To clarify the divergence during cotton evolution, we analyzed the orthologous *CIPK* genes between *G. raimondii*, *G. arboreum*, and the A and D subgenomes of the two allotetraploid species (*G. hirsutum* and *G. barbadense*) (Table S5, Figure S1). In *G. hirsutum*, a total of 37 *CIPK* genes in the D subgenome had orthologs in the D genome, and 35 genes in the A subgenome had orthologs in the A genome. In *G. barbadense*, 34 *CIPK* genes in the A subgenome had orthologs in the A genome, while 40 genes in the D subgenome had orthologs in D genome. This revealed that the collinear relationship of *CIPK* genes between Dt subgenome and D genome was higher than the At subgenome and A genome, suggesting that more *CIPK* genes were lost in the At subgenome of *G. hirsutum* or *G. barbadense* during evolution. A total of 71 *CIPK* genes in *G. hirsutum* had orthologs in the *G. barbadense*, and 39 pairs of orthologous genes between *G. raimondii* and *G. arboreum* (Table S5, Figure S1). Additionally, a total of 39 and 38 pairs of homoeologs for *CIPK* genes was identified in *G. hirsutum* and *G. barbadense*, respectively (Table S6, Figure S2), since each pair had one gene from the At subgenome and another from the Dt subgenome.

### 2.2. Phylogenetic Classifications, Structural Features, and Conserved Motifs Analysis of Cotton CIPK Genes

**Figure 1:**
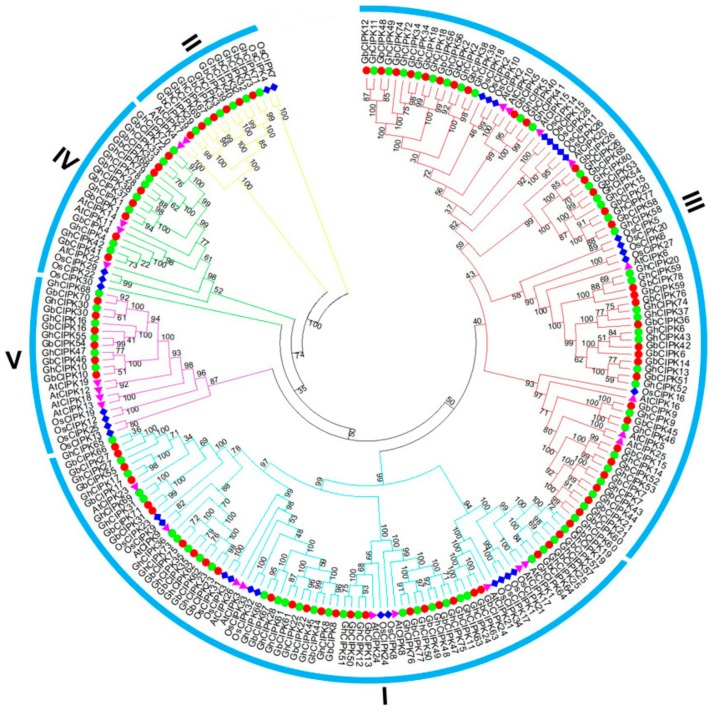
Phylogenetic relationships of calcineurin B-like protein-interacting protein kinases (CIPKs) from *G. hirsutum*, *G. barbadense*, *Arabidopsis*, and rice. The unrooted phylogenetic tree was constructed with MEGA 5.0 using the neighbor-joining method, and bootstrap analysis was performed with 1000 replicates. The CIPKs from *G. hirsutum*, *G. barbadense, Arabidopsis*, and rice are marked with green dots, red dots, magenta triangles, and blue rhombus, respectively.

The evolutionary relationship of *CIPK* genes from *G. hirsutum* (80), *G. barbadense* (78), *Arabidopsis* (26), and rice (34) is shown in Figure 1. A total of 218 *CIPKs* was divided into five groups (I, II, III, IV, and V), consistent with previous reports of *CIPKs* in *Arabidopsis* and poplar [12]. Group I consisted of 55 cotton *CIPK* genes (28 *G. hirsutum* and 27 *G. barbadense CIPK* genes), as compared to 9 *Arabidopsis* and 12 rice *CIPK* genes. Groups II and V contained an equal number of cotton *CIPK*s, with 6 *G. hirsutum* and 6 *G. barbadense CIPK* genes, as compared to 2 and 4 *CIPK* genes for Groups II and V, respectively, each from *Arabidopsis* and rice. Group III is the largest and was composed of 84 *CIPK* genes, including 32 from *G. hirsutum*, 31 from *G. barbadense*, 8 from *Arabidopsis*, and 13 from rice. Group IV contained 8 *G. hirsutum* and 8 *G. barbadense CIPK* genes, as compared to 3 *CIPK* genes each from *Arabidopsis* and rice. In the phylogenetic tree, *G. hirsutum* and *G. barbadense* contained more *CIPK* genes than *Arabidopsis* and rice in each group, and cotton *CIPK* genes had closer relationships with *AtCIPKs* than with *OsCIPKs*, which is consistent with our understanding of the evolutionary history of these genes in plants. From the view of evolution, one *CIPK* gene in *G. raimondii* corresponds with one orthologous gene in *G. arboreum* and two orthologous genes in *G. hirsutum* and *G. barbadense* (Figures S3 and S4), since the two diploid genomes of *G. raimondii* and *G. arboreum* are the progenitors for *G. hirsutum* and *G. barbadense* [27]. Most *CIPK* genes exhibit such a phylogenetic correspondence in *G. arboreum*, *G. raimondii*, *G. hirsutum*, and *G. barbadense.* As highlighted in Figure S3, two *CIPK* genes (*GrCIPK7* and *GrCIPK39*) from *G. raimondii* showed a one-to-one correspondence with two (*GhCIPK63* and *GhCIPK71*) in the D subgenome of *G. hirsutum.* However, there were no corresponding *CIPK* genes in *G. arboreum* for the two genes (*GhCIPK24* and *GhCIPK32*) from A subgenome of *G. hirsutum*, suggesting that two genes were lost in *G. arboreum* after the divergence from *G. raimondii*. Futhermore, we observed *GaCIPK12* and *GrCIPK18* lost corresponding genes in *G. barbadense*, and there was no corresponding gene in *G. raimondii* for *GbCIPK7* in the D subgenome of *G. barbadense*, and a similar phenomenon was also observed in *GbCIPK24* (Figure S4). Additionally, three genes (*GaCIPK12*, *GaCIPK19*, and *GaCIPK37*) in *G. arboreum* only had corresponding genes in *G. hirsutum*, and *GrCIPK32* only had one corresponding gene in *G. barbadense* (Figures S3 and S4).

**Figure 2:**
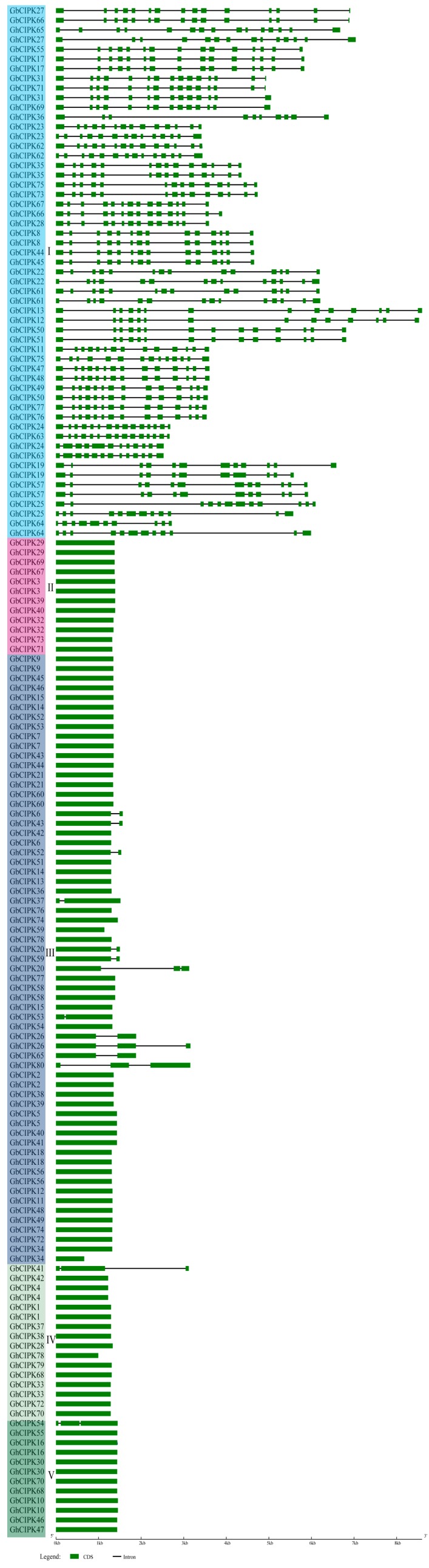
The gene structure of *CIPK* genes in *G. hirsutum* and *G. barbadense*. Introns are presented by black lines and exons by green boxes.

We further analyzed the *CIPKs* gene structure from *G. hirsutum* and *G. barbadense* (Figure 2). Our results show that these *CIPK* genes are clearly divided into an exon-rich clade (>9 exons per gene) and an exon-poor clade (<3 exons per gene). The exon-rich clade members containing 10 to 16 exons were clustered to Group I, while the exon-poor clade members containing 1 to 3 exons were clustered to the other four groups (Group II, III, IV, and V; Figure 2). Gene length in the exon-rich clade was longer than that in the exon-poor clade. The code length of gene members in the exon-rich clade ranged from 4355 to 8597 bp, while in the exon-poor clade, it ranged from 1223 to 2799 bp, except for *GhCIPK34*, *GhCIPK78*, *GbCIPK41*, *GbCIPK20*, *GhCIPK80*, and *GhCIPK26*, which contained 662, 992, 3117, 3127, 3154, and 3156 bp, respectively. Thus, consistent with the phylogenetic tree in Figure 1, the gene structures of *GhCIPKs* and *GbCIPKs* were highly similar in the same group. For example, all the cotton *CIPK* genes (6 *GbCIPKs* and 6 *GhCIPKs*) in Group II contained only one exon, and 15 of 16 cotton *CIPK* genes (7 *GbCIPKs* and 8 *GhCIPKs*) in Group IV contained one exon.

Conserved motifs were also scanned in these CIPK proteins using the MEME online tool. A total of 18 motifs were identified (Figure S5), and the details of each motif are shown in Figure S6. All cotton CIPK proteins contained motif 13, which was annotated as the NAF domain, except GhCIPK36. GhCIPK5, GhCIPK41, GbCIPK5, and GbCIPK40 did not present motif 13, but they contained the amino acid sequence of the NAF domain (Figure S7). Additionally, motifs 16 and 17 were only presented in Group II, suggesting that they might perform group specific functions.

### 2.3. Chromosomal Location and Gene Duplication of CIPK Genes in Two Gossypium Species

**Figure 3:**
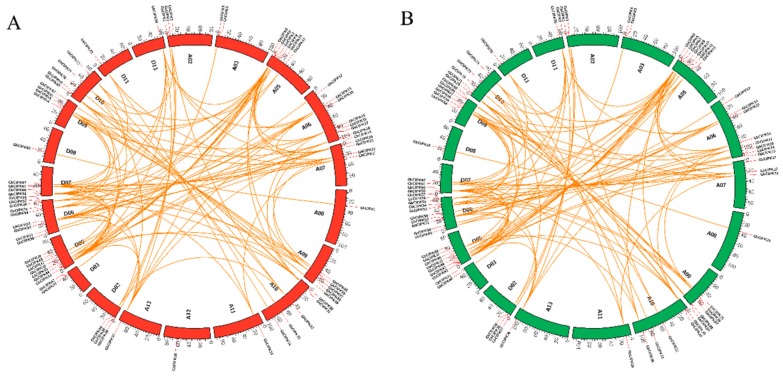
Genomic localization and gene duplication of *CIPK* genes in *G. hirsutum* and *G. barbadense*. (**A**) Genomic localization and gene duplication of *CIPK* genes in *G. hirsutum*; (**B**) Genomic localization and gene duplication of *CIPK* genes in *G. barbadense*. Each of the duplicated gene pairs in *GhCIPKs* and *GbCIPKs* is connected with orange color lines. Chromosomes in *G. hirsutum*and *G. barbadense* are marked with red and green, respectively.

The 80 *GhCIPK*s were mapped to their corresponding chromosomes (Table S2, Figure 3A), and all of them were unevenly distributed on the chromosomes of *G. hirsutum*. For example, all 80 *GhCIPKs* were distributed on 21 of 26 chromosomes, except for A01, A04, D01, D04, and D12. Chromosome D06 contained the largest member (9) of *GhCIPKs* among all upland cotton chromosomes, while chromosomes A11, A12, A13, D11, and D13 each contained only one *GhCIPK* gene (Figure 3A). We also mapped the *GbCIPKs* from *G. barbadense* and found that all 78 *GbCIPKs* were also unevenly distributed on their chromosomes (Table S3, Figure 3B). All 78 *GbCIPKs* were found on 20 of 26 chromosomes, and D06 also contained the largest member (10) of *GbCIPKs*, followed by A06 with eight *GbCIPKs* (Figure 3B).

To elucidate the driving force for the evolution and the functional divergence of *CIPK* genes, the occurrence of duplication events of cotton *CIPK* genes was analyzed. As shown in Figure 3 and Table S7, 109 and 107 duplicated gene pairs of *CIPK* genes were identified in *G. hirsutum* and *G. barbadense*, respectively. All were involved in segmental duplication, since these duplicated gene pairs were located on different chromosomes. In addition, there were a series of several duplication events in the two cotton species, for example, *GhCIPK11/GhCIPK49*, *GhCIPK11/GhCIPK72*, *GbCIPK34/GbCIPK48*, and *GbCIPK34/GbCIPK74*. These results suggest that segmental duplication events contributed to the *CIPK* gene family in both of *G. hirsutum* and *G. barbadense*. We further calculated the Ka/Ks ratio to explore the selective constraints on each pair of duplicated *CIPK* gene in *G. hirsutum* and *G. barbadense* (Table 1). In *G. hirsutum*, 85 of 109 duplicated *GhCIPK* gene pairs had a Ka/Ks ratio <1, which demonstrates that these genes had undergone strong purifying selection pressure; one gene pair (*GhCIPK9/GhCIPK46*) had a Ka/Ks ratio >1, indicating that this gene might have undergone positive selection; the remaining 24 duplicated pairs with ratios =1 seemed to be under neutral selection. In *G. barbadense*, almost all *GbCIPK* duplicated pairs had a Ka/Ks ratio <1, three pairs (*GbCIPK8/GbCIPK44*, *GbCIPK10/GbCIPK46*, and *GbCIPK36/GbCIPK76*) had a Ka/Ks ratio >1, and three pairs (*GbCIPK13/GbCIPK50*, *GbCIPK14/GbCIPK51*, and *GbCIPK59/GbCIPK78*) had a Ka/Ks ratio = 1. These results suggested that the function of the duplicated *CIPK* genes in *G. hirsutum* and *G. barbadense* did not diverge much during subsequent evolution, and purifying selection could mainly contribute to the maintenance of function in the two *Gossypium CIPK* gene families.

### 2.4. Analysis of Cis-Elements and Prediction of Transcription Factor Binding Sites in the Promoter Regions of GhCIPKs

Analysis of *cis-*elements in genes could provide critical evidence for the gene’s function. Many studies reported that *CIPK* genes play key roles in various stresses [17,22,25]. Here, the putative stress-related and hormone-related *cis-*elements were scanned in the 1.5 kb upstream of the start codons of 80 *GhCIPKs* using the PlantCARE database. In total, 12 stress-related and hormone-related *cis-*elements were predicted in the promoters of 80 *GhCIPKs* (Table S8). Among those *GhCIPKs*, 36 *GhCIPKs* had the abscisic acid (ABA) responsive element (ABRE), 8 *GhCIPKs* harbored the auxin responsive element (TGA-element and AuxRR-core), 17 *GhCIPKs* contained defense and stress responsive elements (AT-rich and TC-rich repeats), 16 *GhCIPKs* contained the drought responsive element (MBS), 25 *GhCIPKs* harbored the gibberellin (GA) responsive element (TATC-box, P-box, and GARE-motif), 22 *GhCIPKs* had the salicylic acid (SA) responsive element (TCA-element and SARE), 81 *GhCIPKs* possessed the light responsive element (G-box, Box 4, GT1-motif, MRE, AE-box, ACE, Sp1 and I-box), 45 *GhCIPKs* had the methyl jasmonate (MeJA) responsive element (TGACG-motif and CGTCA-motif), 45 *GhCIPKs* contained the anaerobic induction responsive element (ARE), 19 *GhCIPKs* possessed the low temperature-responsive element (LTR), 25 *GhCIPKs* had the wound-responsive element (WUN-motif), and 15 *GhCIPKs* contained the wounding and pathogen responsive element (W box). In total, 68 *cis-*elements were related to ABA, 10 to auxin, 19 to defense and stress, 23 to drought, 33 to GA, 22 to SA, 341 to light, 66 to MeJA, 64 to anaerobic induction, 22 to low-temperature, 31 to wound-responsive, and 15 to wounding and pathogen were identified in the 80 *GhCIPKs*. These results suggest that *GhCIPKs* could be involved in various regulatory mechanisms when cotton plants are subjected to various stresses.

It is well known that TFs regulate the transcription of their target genes by binding certain upstream elements [39]. To explore the regulatory interactions between TFs and *GhCIPKs*, we further searched highly conserved transcription factor binding sites (TFBSs) in 1.5 kb promoter regions of the 80 *GhCIPKs*. The results showed that 166 TFs belonging to 28 families might bind to the 1.5 kb promoter regions of 80 *GhCIPKs* (Table S9). Among the 28 TF families, many were found in plant-specific families, such as the B3, G2-like, NAC, TCP, and LBD. However, many TFs were also found in animals, bacteria, and yeast, such as the ERF, C2H2, HD-ZIP, MYB and TALE families. Numerous reports emphasize that these TF families play key roles in plant responses to environmental stimuli and regulation, such as seed storage protein synthesis, carbohydrate metabolism, plant defense mechanisms, seed germination, hormonal signal transduction and response to various biotic and abiotic stresses [40,41,42,43].

### 2.5. Expression Profiling of GhCIPK Genes in Different Tissues and Under Various Stresses

**Figure 4:**
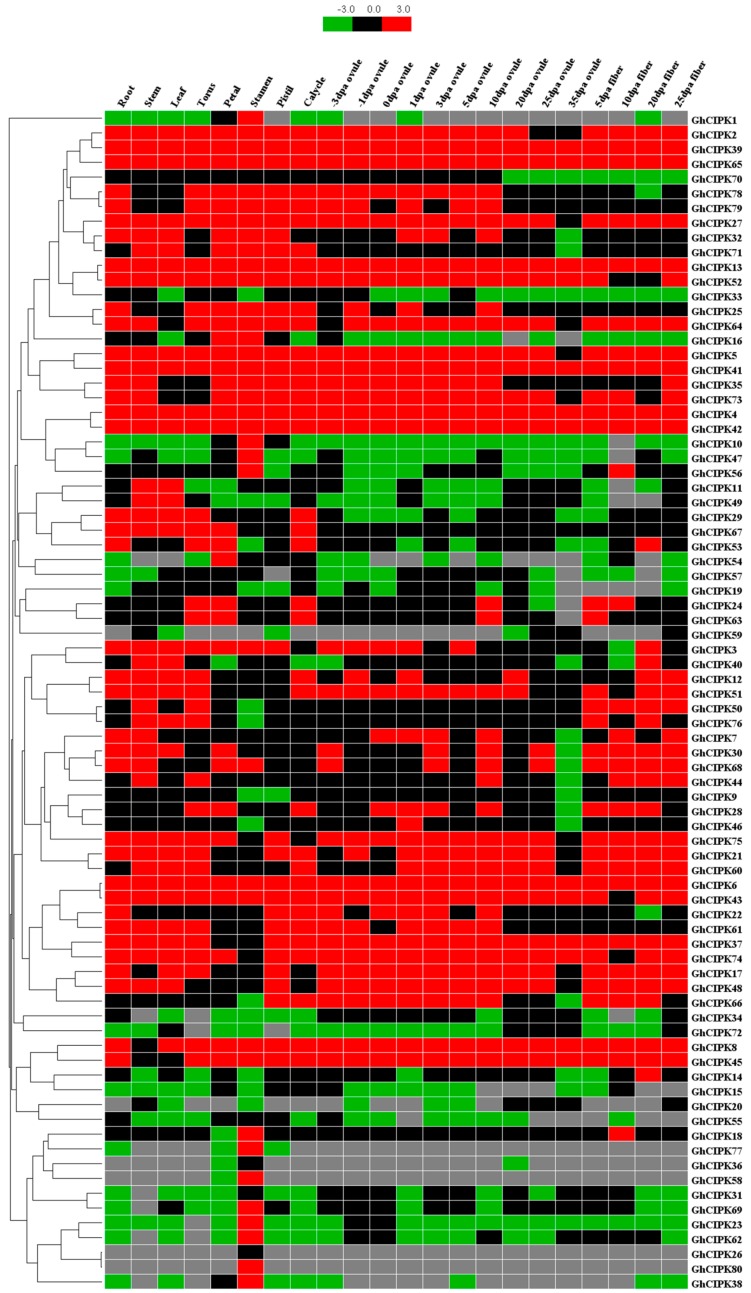
Expression patterns of 80 *ChCIPK* genes in different tissues of TM-1. The raw data for RNA-Seq of *G. hirsutum* acc. TM-1 [34] was downloaded and used to analyze the expression patterns of *ChCIPK* genes. The color bar represents the expression values in log2 of fragments per kilobase per million reads (FPKMs). The gray color represents that there is no expression.

To understand the possible functions of *GhCIPK* genes in different tissues, the gene expression patterns of 80 *GhCIPK* genes from the public RNA-seq data of TM-1 were analyzed in different tissues (root, stem, leaf, cotyledon, torus, petal, stamen, pistil, and calycle) and developmental stages (ovule and fiber) (Figure 4). As shown in Figure 4, 7 *GhCIPK*s were highly expressed in all tested tissues of TM-1, including *GhCIPK4*, *GhCIPK6*, *GhCIPK13*, *GhCIPK39*, *GhCIPK41*, *GhCIPK42*, and *GhCIPK65*; *GhCIPK72* was expressed at low levels in all tested tissues except torus and pistil. This indicated that some *GhCIPK* genes had multiple biological functions during cotton development. Additionally, several *GhCIPK* genes were specifically expressed in one or several specific tissues. For example, *GhCIPK58* was only expressed in petals and stamens. *GhCIPK26* and *GhCIPK80* were only expressed in stamens, while *GhCIPK1*, *GhCIPK10*, and *GhCIPK47* were highly expressed in stamens than in other tissues. These results imply that these genes play key roles in the development or function of specific tissues.

**Figure 5:**
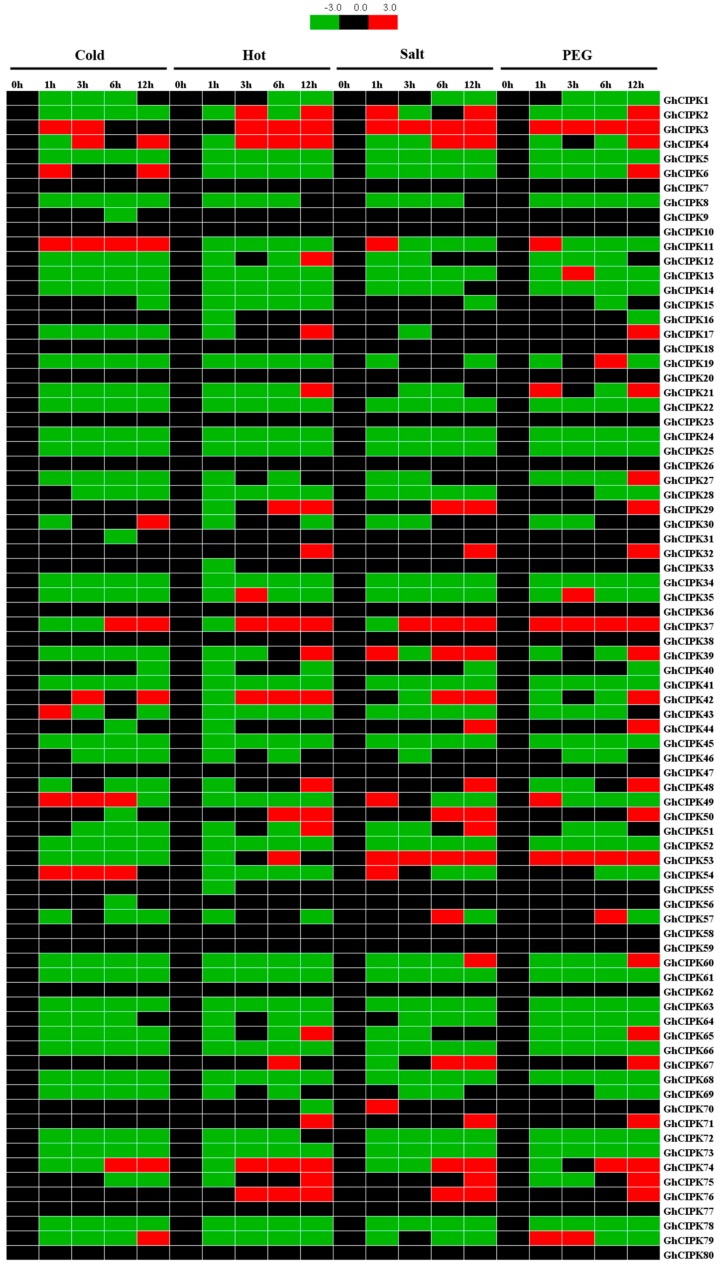
Expression patterns of 80 *ChCIPK* genes in response to various stresses. The colored bar represents the relative expression levels.

Previous studies reported that *CIPK* genes could play important roles in plant responses to various stresses [14,22,25,44]. Therefore, we analyzed the expression patterns of the 80 *GhCIPKs* under various stresses, including cold, hot, salt, and drought stresses, using the published RNA-seq data of TM-1. As shown in Figure 5, the relative expression levels of some *GhCIPK*s were either induced or suppressed by these four treatments. There were 16 *GhCIPKs* downregulated under the four stress treatments. The expression levels of *GhCIPK3* and *GhCIPK53* were significantly upregulated under salt or drought stress. The expression of *GhCIPK11* was significantly upregulated under cold stress, and *GhCIPK37* was upregulated under drought stress. Several *GhCIPKs* were upregulated at only one time point in at least one treatment. For example, *GhCIPK13* was upregulated at 3 h under drought stress, while *GhCIPK48* and *GhCIPK71* were upregulated at 12 h under hot, drought, or cold stress.

**Figure 6:**
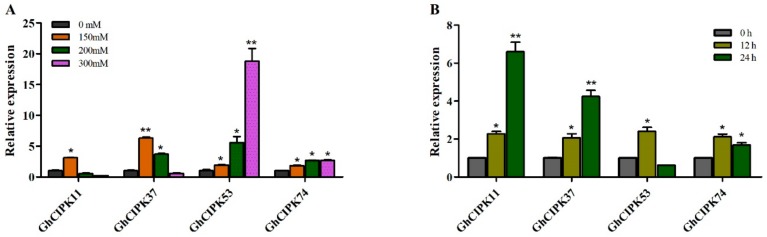
Relative expression levels of four *ChCIPK* genes under salt and cold treatments. (**A**) Relative expression levels of four *ChCIPK* genes under salt treatment; (**B**) relative expression levels of four *ChCIPK* genes under cold treatment. The *GhUBQ7* was used as an internal control. Error bars indicate the standard deviations of three independent experiments. Significant differences are determined by *t*-test compared to control treatment (* *p* < 0.05; ** *p* < 0.01).

To further confirm the expression patterns of *GhCIPK* genes in response to environmental stresses, we selected four *GhCIPK* genes to examine their expression profiles under salt stress and cold stress. As shown in Figure 6, *GhCIPK53* and *GhCIPK74* showed similar expression patterns, which were upregulated by different salt treatments. The expression level of *GhCIPK11* was only upregulated at 150 mM salt treatment, while *GhCIPK37* was upregulated at 150 mM and 200 mM salt treatments, respectively. Under cold stress, three genes (*GhCIPK11*, *GhCIPK37*, and *GhCIPK74*) were significantly upregulated; the expression level of *GhCIPK53* quickly peaked after 12 h and then decreased at 24 h of cold stress. These results suggest that *GhCIPK* genes might enhance the adaptability of cotton to various abiotic stresses.

### 2.6. Co-Localization and Sequence Variation of GhCIPK Genes with QTLs for Seed Oil and Protein Content

Although there are many studies in genetic analysis and QTL mapping of oil content in different cotton populations [26,28], the genetic relationships of cotton *CIPK* gene family with oil and protein content has not been reported on cotton. Here, we co-localized the *GhCIPK* genes with reported quantitative trail loci (QTLs) for oil and protein contents. The QTLs of intraspecific *G. hirsutum* and interspecific *G. hirsutum* × *G. barbadense* populations were downloaded from the CottonQTL database [45,46] and a recently published article [47]. Finally, 14 *GhCIPK* genes were mapped with the anchored cotton oil QTL or protein QTLs within a 20 cM region (Figure S8). There were two genes (*GhCIPK4* and *GhCIPK5*) on chromosome A03/c3, two genes (*GhCIPK13* and *GhCIPK14*) on A06/c6, and one gene (*GhCIPK37*) on A13/c13 located within oil or protein content QTLs in the At subgenome of *G. hirsutum*. Two genes (*GhCIPK41* and *GhCIPK42*) on chromosome D03/c14, two genes (*GhCIPK47* and *GhCIPK48*) on chromosome D05/c19, two genes (*GhCIPK61* and *GhCIPK62*) on chromosome D07/c16, two genes (*GhCIPK65* and *GhCIPK66*) on chromosome D09/c23, and one gene (*GhCIPK74*) on chromosome D13/c18 were located within the oil or protein QTLs in the Dt subgenome of *G. hirsutum*. 

We further identified sequence variation of the 14 *GhCIPKs* by scanning the RNA-seq data of CRI 36 and Hai 7124. Finally, 17 SNPs, classified as non-synonymous, were identified from the seven *GhCIPKs* (Table S10). Additionally, these SNPs were further analyzed in the five published cotton genome data (TM-1_NAU, TM-1_ZJU, 3-79, Xinhai 21 and Hai 7124), and nine SNPs of four *GhCIPKs* were confirmed as non-synonymous mutations. Primers of the nine SNPs were designed (Table S11) and screened in a backcross inbred line (BIL) population of 180 lines derived from a backcross between CRI 36 and Hai 7124 as described previously [48]. Five SNPs from four *GhCIPKs* were found to be significantly associated with oil content in the BIL population (Table S12). For example, in the BIL population, *GhCIPK14-1273* (T/G) and *GhCIPK42-340* (T/C) resulted in a change from cysteine to glycine and from phenylalanine to leucine, respectively; the two SNPs were found to be significantly associated with oil content in 2016XJ (−0.197 and −0.182). *GhCIPK47-1318* (A/C) and *GhCIPK66-917* (G/A) resulted in lysine to glutamine and a glycine to glutamic acid, respectively. The two SNPs were also found to be significantly associated with oil content in 2016AY (−0.162, −0.156). *GhCIPK66-406* (T/G), which resulted in a tyrosine to aspartic acid change, was significantly associated with oil content in 2015AY (−0.209). However, the five SNPs were only associated with oil content in one test, and the association between the five SNPs and oil content needs further studies. These results show that most *GhCIPK* genes were not associated with natural variations in cotton oil content.

**Figure 7:**
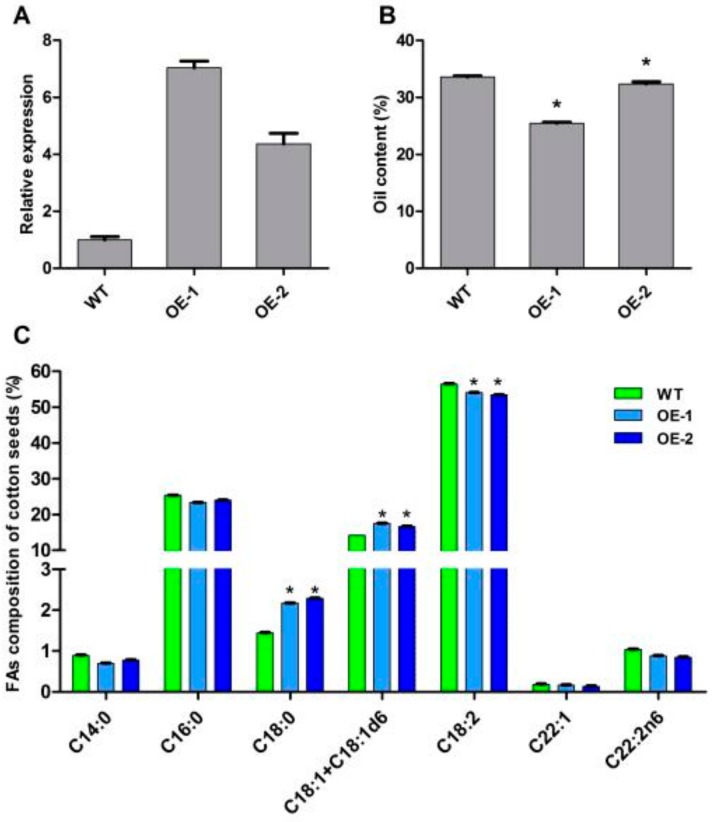
Overexpression of *GhCIPK6* changed the oil content and fatty acid composition in transgenic cotton. (**A**) Real-time qRT-PCR analysis of *GhCIPK6* expression in transgenic and wild type (WT) cotton plants. OE-1 and OE-2 were different transgenic lines; (**B**) cottonseed oil content of transgenic and WT cotton plants. Error bars indicate the standard error. Asterisks indicated significant differences (*p* < 0.05, *) between transgenic and WT plants; (**C**) fatty acid composition in cottonseeds of transgenic and wild type (WT) plants. The student’s t-tests was used to evaluate significant difference (*p* < 0.05, *).

One recent report showed that the overexpression of *BnCIPK9* during seed development reduced seed oil content in transgenic *B. napus*, and the seed oil content of *Atcipk9* mutants was significantly higher than that of WT plants [29]. To confirm whether *GhCIPK* genes affect cottonseed oil content, the plasmid of recombinant vector (pBI121-GhCIPK6) was injected into the ovary of ‘11-0516′ by syringe, and putative transgenic cotton seeds of T_0_ were harvested and further grown. Southern blotting revealed that two transgenic cotton plants had two copies, while no hybridizing band was found in wild-type (11-0516) (data not shown). The relative expression levels of *GhCIPK6* in the two T_3_ transgenic cotton lines were higher than that in wild-type plants (Figure 7A). To characterize the biological function of *GhCIPK6* in oil synthesis, the oil content of mature seeds from the two transgenic lines and wild-type plants was detected using an NMI20-Analyst nuclear magnetic resonance spectrometer (Niumag, Shanghai, China). As shown in Figure 7B, the two transgenic lines with increased expression levels of *GhCIPK6* had significantly lower oil content (25.44% and 32.37%) than wild-type plants (33.58%). Furthermore, the relative proportions of C18:1 and C18:1+C18:1d6 from transgenic lines cottonseeds were significantly increased, while the relative proportion of C18:2 was significantly decreased, compared to wild-type plants (Figure 7C). These results indicated that *GhCIPK6* may play a negative role in oil synthesis.

### 2.7. Predictions of Putative Molecular Regulatory Mechanisms of GhCIPKs

The previous sections characterized the roles of *GhCIPKs* in different tissues, various stresses, and oil content. In this section, we characterize post-transcriptional regulation mediated by alternative splicing (AS) and miRNAs to predict the putative molecular regulatory mechanism of *GhCIPK*s in expression and their functional multiplicity.

AS, as a post-transcriptional regulation mechanism in eukaryotes, can generate multiple transcripts from the same gene to increase proteome diversity and regulate mRNA levels [49]. In *Arabidopsis*, it was estimated that more than 60% of exon-containing genes were subject to AS [50]. Many studies showed that more AS isoforms presented specific expression patterns in cells, tissues or different conditions, and the extent of AS was related to the complexity of tissues [51,52]. Based on our mRNA-seq data of cotton floral buds, the potential relationship between potential AS events of the *GhCIPK* genes and cotton floral buds was analyzed. We detected a total of 39 AS events from nine *GhCIPKs* in cotton floral buds, and seven *GhCIPKs* undergoing AS were associated with intron retention (IR) events, and the number of exon skipping (ES) (five *GhCIPKs*) was the second most common of AS events (Figure S9A). We randomly selected one gene (*GhCIPK17*) to validate the accuracy of the AS events using RT-PCR with the corresponding primers (Table S11). We found that each of the amplified fragment sizes was consistent with that of the predicted fragment (Figure S9B); subsequently, the amplified fragments were cloned for Sanger sequencing. Finally, the sequence consistency between cloned fragments and predicted sequences was observed based on our mRNA-seq of cotton floral buds.

**Figure 8:**
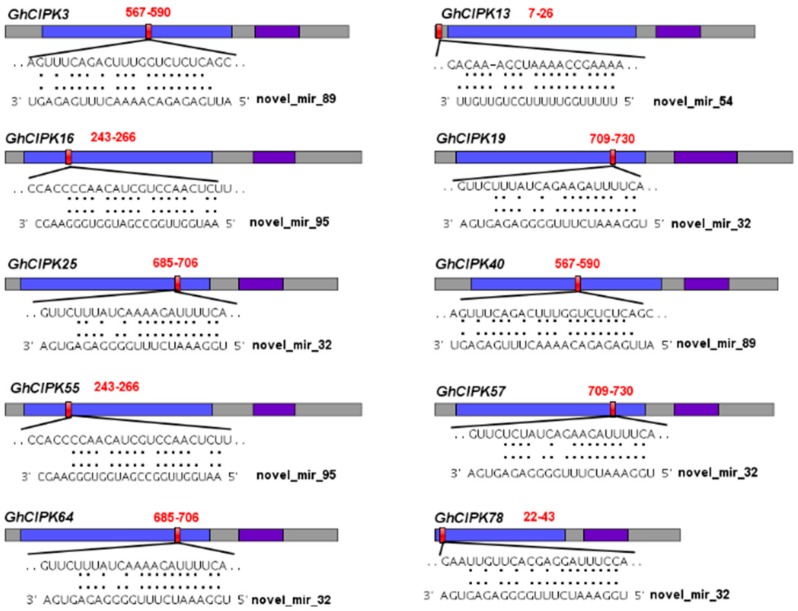
The miRNA-mediated targeting regulatory relationships of *GhCIPKs*. The ORFs (open reading frames) of *GhCIPKs* are indicated by heavy grey boxes. The protein kinase domain (PF00069) and the NAF domain (PF03822) are shown in blue and purple boxes, respectively. miRNA complementary sites with the nucleotide positions of *CIPK* genes are filled by the color red.

In plants, miRNAs regulate gene expression at the post-transcriptional level by mediating mRNA cleavage or translational repression [53]. To explore the mechanisms of *GhCIPK* gene family regulated by miRNA, we searched the coding sequence regions of 80 *GhCIPKs* for putative target sites of cotton miRNA using the psRNATarget server with parameters described in Section 4. A total of 11 upland cotton miRNAs targeted 25 *GhCIPKs* (Figure 8, Table S13). As shown in Figure 8, *GhCIPK3* and *GhCIPK40* were both targeted by novel_mir_89 with sites in the kinase domain. *GhCIPK13* was targeted by novel_mir_54 with sites in the 5′-end of CDS. *GhCIPK16* and *GhCIPK55* were both targeted by novel_mir_95 with sites in the kinase domain. One upland cotton novel_mir_32 regulated *GhCIPK19*, *GhCIPK25*, *GhCIPK57*, *GhCIPK64*, and *GhCIPK78* in the kinase domain. In addition, we also found that *GhCIPK6* and *GhCIPK43* were both targeted by ghr-miR7498 with sites in the NAF domain. One upland cotton novel_mir_27 targeted *GhCIPK26*, *GhCIPK48*, *GhCIPK75*, and *GhCIPK77* in the NAF domain. *GhCIPK11* and *GhCIPK49* were both targeted by ghr-miR7495a with sites in the kinase domain. One ghr-miR7509 targeted *GhCIPK35* and *GhCIPK73* in the NAF domain. Intriguingly, both novel_mir_95 and novel_mir_89 regulated *GhCIPK3*, but had different complementary sites and unrelated sequences. This case was also found in *GhCIPK12*, *GhCIPK19*, and *GhCIPK40*. For example, *GhCIPK19* was targeted by ghr-miR7496a and novel_mir_32 with sites in the NAF domain and the kinase domain, respectively (Table S13). The divergence of miRNA target sites indicated that members of *GhCIPKs* might be regulated by different miRNAs. We further tested the expression patterns of the relationship between miRNAs and *GhCIPK* genes in cotton ovules through qRT-PCR analysis (Figure S10). Three ghr-miRNAs (novel_mir_54, ghr-miR7498, and novel_mir_27) and their target *GhCIPK* mRNAs (*GhCIPK5*, *GhCIPK6*, and *GhCIPK58*) showed negative regulatory relationships in cotton ovules. This implied that some cotton miRNAs may play important roles in cotton ovules by regulating these *GhCIPK* genes.

Nine *GhCIPKs* contained 34 AS events, including alternative 3′ acceptor sites (AA), alternative 5′ donor sites (AD), intron retention (IR), and exon skipping (ES) were detected from mRNA-seq data in cotton floral buds, (Figure S9A). To understand how miRNAs interact with isoforms, we then predicted target sites for these cotton miRNAs using *CIPK* full-length isoforms. A total of nine isoforms from two *GhCIPKs* were identified to be potential targets of two novel miRNAs (novel_mir_27 targeted isoforms from *GhCIPK48* and novel_mir_63 targeted isoforms from *GhCIPK51*) (Figure S11). Furthermore, we examined the effect of AS on gain/loss of miRNA target sites among the nine *GhCIPK* isoforms regulated by miRNAs; the results showed that the miRNA target sites in nine isoforms were not affected by AS events (Figure S11). This result is possibly because only one or two miRNAs regulated the isoforms of the *GhCIPKs* or only part of the isoforms transcribed from *GhCIPK* genes were detected from our mRNA-seq data. Similar phenomena could also be detected in the cotton *CAT* gene family in Figure S6 [39].

## 3. Discussion

### 3.1. Phylogenetic Analysis and Evolution of CIPK Genes in Gossypium

The *CIPK* genes in *G. hirsutum* and *G. barbadense* could be classified into five groups based on the evolutionary tree (Figure 1), similar results have also been found in *Arabidopsis* and *Populus* [12]. As shown in Figure 1, cotton *CIPK* genes were more closely related with *AtCIPKs* than *OsCIPKs*, which was consistent with the evolutionary relationships among cotton, *Arabidopsis*, and rice. Gene structure can provide important information on gene family evolution [54,55]. Cotton *CIPKs* were clearly divided into exon-rich (Group I) and exon-poor (Group II, III, IV, and V) clades based on their gene structures (Figure 2); some similar results for *CIPKs* were also observed in *Arabidopsis*, poplar, and soybean [12,14]. Combined with the evolution analysis of *CIPK* in plants [14], suggests that intron gain or loss events were the major driving factors for the gene structural evolution of the *CIPK* gene family before eudicot-monocot divergence. *CIPK* genes were unevenly distributed on chromosomes of the two allotetraploid cotton species (Figure 3); it indicates that this might be caused by differential rates of genomic evolution and intergenomic hereditary information transfer [56,57].

Allotetraploid cotton species (*G. hirsutum* and *G. barbadense*) resulted from the hybridization between two putative diploid cotton species (A-genome-like African diploid and D-genome-like American diploid) [58]. A total of 80 and 78 *CIPK* genes were identified in *G. hirsutum* and *G. barbadense*, respectively (Tables S2 and S3). The number of *CIPK* genes in each of the *G. hirsutum* and *G. barbadense* genome was greater than that in *Arabidopsis* (26) and rice (34), and was basically equal to the total sum of *G. raimondii* (41) and *G. arboretum* (39), suggesting that the *CIPK* gene family expands during evolution. *G. raimondii* and *G. arboretum* underwent a *Gossypium*-specific whole-genome duplication (WGD) [27,30,31], and the *CIPK* gene family of *G. raimondii* and *G. arboretum* may expand by an ancient WGD event. Therefore, the expansion of the *CIPK* gene family in *G. hirsutum* and *G. barbadense* may be due to the hybridization and subsequent polyploidization event. Meanwhile, duplicated *CIPKs* in *G. hirsutum* and *G. barbadense* were located on different chromosomes and segmental duplication events were important for the expansion of the *CIPK* gene family in *G. hirsutum* and *G. barbadense* (Figure 3 and Table S7). Therefore, we speculated that the expansion of the *CIPK* gene family in the two cotton species was mainly due to WGD/segmental duplications. Gene duplication plays a key role in the process of gene family expansion, and plants rapidly adapt to new environments after segmental duplication and translocation [59]. Most of the duplicated *CIPK* genes in *G. hirsutum* and *G. barbadense* were driven by purifying selection as indicated by the Ka/Ks ratio <1 (Table 1), suggesting that the functions of the duplicated cotton *CIPK* genes were highly conserved during subsequent evolutionary events, which could eliminate deleterious loss-of-function mutations, and a new duplicated gene at both duplicate loci could be fixed and enhanced after purifying selection [60].

### 3.2. Expression Patterns of GhCIPK Gene Family and the Role of GhCIPK6

*CIPK* genes are involved in plant growth and development, oil synthesis, and response to various stresses [16,17,29]. In many plants, the spatiotemporal expression profiles of *CIPK* genes have already been reported, including apple, cassava, *Arabidopsis*, *B. napus*, and wheat. The *MdCIPK* genes were highly expressed in root, flower, and fruit [16]. Additionally, some *CIPK* genes were expressed abundantly in certain tissues (root, flag leaf) in wheat [18]. In the present study, some *GhCIPK* genes were constitutively expressed in our tested tissues, and some *GhCIPK* genes were expressed abundantly in certain tissues (Figure 4), indicating that these genes may play important roles in the growth and development of cotton plants. It is worth noting that 39 pairs of homoelogs for *GhCIPK* genes showed very similar gene structure in terms of exon number and intron length (Figure 2). Among the 109 duplicated gene pairs in *G. hirsutum*, 69 pairs of duplicated *GhCIPKs* showed similar expression patterns (Figure 4), while the expression patterns of others were divergent. The differential expression patterns of duplicated *CIPK* genes suggests that they may have experienced functional divergence. After gene duplication, the new gene could be considered a redundant gene compared with the existing gene, and it can be considered a driving force for evolutionary innovation [61]. Inserting or deleting tissue-specific enhancers or repressors in the coding regions of duplicated genes could obtain a new regulatory context, which might be the cause of functional divergence [62]. Overexpression of *GhCIPK6* in cotton could reduce oil content, and increased C18:1 and C18:1+C18:1d6 in transgenic cotton lines, as compared to wild-type plants (Figure 7), indicated that *GhCIPK6* plays a negative role in oil synthesis. Similar results could also be presented in the function of *SNF1, BnCIPK9*, and *AtCIPK9* [5,29].

### 3.3. Putative Molecular Regulatory Mechanisms of GhCIPKs in Cotton

In the present study, we predicted the TFBSs in the 1.5 kb promoter sequence regions of 80 *GhCIPKs* and found that the 28 different TFs families (Table S9) might bind to *GhCIPK*s and potentially regulate *CIPK* genes’ expression. TFs regulate transcriptional initiation and control multiple cellular processes and are fundamental in plant development and environmental response [63]. In wheat, Luang et al. found that a *SnRK3/CIPK* family member could interact with *TabZIP2*, a *bZIP* TF, and promote its activity [64]. Recently, an integrative picture was drawn, which showed that different SnRKs and TOR kinase were highly interconnected to control nutrient and stress responses of plants [65]. In apple, an apple CIPK protein kinase, MdCIPK22, targeted a novel residue of AREB transcription factor, Thr411, for ABA-induced phosphorylation, and in the ABA signaling pathway, this was a novel phosphorylation site in the CIPK-AREB regulatory module [66]. In *Arabidopsis*, abscisic acid repressor 1 (ABR1) was identified as the downstream target of *CIPK3*, and *CIPK3* interacted with ABR1 to regulate ABA response during seed germination [67]. However, how cotton *CIPK* genes are regulated by these TFs involved in various stresses or plant growth and development remains unknown; this needs to be investigated through the following research using newly developed technologies.

Protein-coding genes may be negatively regulated by a type of miRNA, which plays an important role in a variety of processes, such as response to environmental stress and plant growth and development [53,68,69]. Many miRNAs were identified in cotton, and some of them were differentially expressed in different tissues or under various abiotic stresses [68,70,71]. For example, 65 conserved miRNA families were identified from small RNA-seq of cotton leaf and ovule, and 32 families were expressed differentially between cotton leaf and ovule [68]. A total of 113 miRNAs, containing 111 miRNAs and two novel miRNAs, were identified in Xuzhou 142 and its fuzzless/lintless mutant in 0–10 DNA ovules [72]. Overexpression of miR157 could suppress the expression of SQUAMOSA promoter-binding protein-like (SPL) genes and lead to smaller floral organs, fewer ovules, and decreased seed production [73]. In cotton, 319 known miRNAs and 800 novel miRNAs were identified under low- and high-temperature stresses, and almost one-third of the temperature-responsive miRNA-targeted TFs were shown to be related to the regulation of plant growth and development, including the *CIPK* family [74]. However, there remains a lack of information about miRNA-guided regulation of cotton *CIPK* genes at the post-transcriptional level in cotton growth and development. In our study, we found three ghr-miRNAs (novel_mir_54, ghr-miR7498, and novel_mir_27) and their target *GhCIPK* mRNAs (*GhCIPK5*, *GhCIPK6*, and *GhCIPK58*) showed negative regulatory relationships in cotton ovules by qRT-PCR (Figure S10). This implies that the three cotton miRNAs may play important roles in cotton ovules by regulating *GhCIPK5*, *GhCIPK6*, and *GhCIPK58*, respectively. Our findings suggest that some *GhCIPKs* could be regulated by upland cotton miRNAs, but the true regulatory relationships between miRNAs and *GhCIPKs* need further study.

## 4. Materials and Methods

### 4.1. Identification of CIPK Genes in G. hirsutum and G. barbadense

Genome assemblies of *G. hirsutum* acc. TM-1 from Nanjing Agricultural University (NAU version), Zhejiang University (ZJU version,) and Huazhong Agricultural University (HAU version) were downloaded from the Cotton Functional Genomics Database (CottonFGD) (https://cottonfgd.org/) [75], and three genome files of *G. barbadense*, acc. 3-79 (HAU version), cv. Xinhai 21 (NAU version), and cv. Hai7124 (ZJU version) were also downloaded from CottonFGD (https://cottonfgd.org/). The published CIPK proteins of *Arabidopsis* [9] and rice [10] were downloaded from the TAIR (https://www.arabidopsis.org/index.jsp) database and the rice genome database (http://rice.plantbiology.msu.edu//), respectively (Table S1). Afterwards, they were used as queries to search against *G. hirsutum* and *G. barbadense* genome databases with BLASTP program with an *E*-value < 1 × 10^−5^. Then, each cotton CIPK protein was subjected to the InterProscan (http://www.ebi.ac.uk/Tools/pfa/iprscan/) [76] and SMART (http://smart.embl-heidelberg.de/) [77] databases to confirm the presence of the protein kinase domain (PF00069) and the NAF domain (PF03822). The details on *GhCIPK* and *GbCIPK* genes were obtained from CottonFGD (https://cottonfgd.org/).

### 4.2. Phylogenetic Analysis and Synteny Analysis

The protein sequences of all the identified CIPKs from *G. hirsutum, G. barbadense*, *Arabidopsis*, and rice were aligned using the Clustal X v2.0 program [78]. A phylogenetic tree was constructed using the neighbor-joining (NJ) method in MEGA 5.0 software [79] with poisson correction model. The classification of *GhCIPKs* and *GbCIPKs* was consistent with the previous classification reported in *Arabidopsis* and poplar [12]. The gene structures of cotton *CIPK*s were graphically visualized using the GSDS (Gene Structure Display Server) tool [80]. The MEME program (http://meme-suite.org/tools/meme) was used to analyze the motifs of cotton CIPK proteins. The number of repetitions was set as any, the optimum width of motifs ranged from 6 to 200 residues, the maximum number of motifs was 18, and the other default parameter settings were used.

The chromosomal location images of cotton *CIPK*s were visualized with Mapchart v2.2 [81] and Circos-0.67 (http://circos.ca/). Orthologous and homoeologs for *CIPK* genes were identified among the genomes of the diploid and allotetraploid cotton species based on phylogenetic trees and sequence alignments [48,82]. The gene duplication events of two allotetraploid cotton species were analyzed by using MCScanX software, and according to the length of aligned sequence, covered >80% between aligned gene sequences, similarly to the aligned regions (>80%) [48]. DnaSP v5.0 software [83] was used to calculate the synonymous substitution (Ks) and nonsynonymous substitution (Ka).

### 4.3. Prediction of Cis-Elements and Transcription Factor Binding Sites in the Promoter Region

The genomic sequences 1.5 kb upstream of the start codons of 80 *GhCIPK*s were extracted, and their regulation elements were predicted using the PlantCARE database (http://bioinformatics.psb.ugent.be/webtools/plantcare/html/). The putative transcription factor binding sites (TFBSs) of the *GhCIPK* gene promoter regions were predicted using the PlantTFDB 4.0 server (http://planttfdb.cbi.pku.edu.cn/) with a stricter parameter: threshold *p*-value ≤ 1 × 10^−7^.

### 4.4. Alternative Splicing Events Analysis and Potential microRNA Target Analysis

Alternative splicing (AS) events of *GhCIPKs* were identified from our mRNA-seq data of cotton floral buds, and AS events were classified into alternative 3′ acceptor sites (AA), alternative 5′ donor sites (AD), intron retention (IR), and exon skipping (ES).

Upland cotton microRNA (miRNA) sequences were obtained from the plant MicroRNA database (http://bioinformatics.cau.edu.cn/PMRD/), miRBase (http://www.mirbase.org/) and published articles [53,70]. *GhCIPK* genes targeted by miRNAs were predicted by searching the coding sequence regions of 80 *GhCIPKs* for complementary sequences using the online psRNATarget server (http://plantgrn.noble.org/psRNATarget/home) with default parameters as described in a reference [78].

### 4.5. Plant Growth Conditions and Treatments

Cotton seedlings of TM-1 were grown in a plant growth chamber at 28 ± 2 °C under a 16 h light/8 h dark cycle. The third true leaf stage seedlings were treated with salt stress or cold stress. Seedlings were grown in Hoagland nutrient solution and treated with 150, 200, and 300 mM NaCl for 24 h and treated with water for 24 h as a control condition. Seedlings were incubated at 4 °C for 0, 12, and 24 h in a temperature-controlled chamber. The leaves at each time point of each treatment were harvested and immediately frozen with liquid nitrogen and stored at −80 °C for subsequent RNA isolation.

### 4.6. RNA Isolation and Expression Profiling Analysis

The RNAprep Pure Plant Kit (Tiangen, Beijing, China) was used to extract total RNA from all samples. Approximately 500 ng of RNA was reverse transcribed to cDNA using the PrimerScript 1st Strand cDNA Synthesis Kit (TaKaRa, Dalian, China). Reverse transcription polymerase chain reaction (RT-PCR) was used to validate the accuracy of AS events, and the reaction was performed at 50 °C for 30 min, 95 °C for 2 min, followed by 29 cycles at 94 °C for 30 s, 60 °C for 30 s, and 72 °C for 35 s, followed by 72 °C for a 2 min extension step. The gene-specific primers for qRT-PCR and RT-PCR were designed using Primer v5.0 software, and primers for RT-PCR were designed to span the splicing events. All primers are listed in Table S11. *GhUBQ7* was used as an internal control. The qRT-PCR was strictly performed with SYBR Premix Ex Taq Kit (TaKaRa) according to the manufacturer’s instructions, and qRT-PCR was performed at 95 °C for 2 min, followed by 40 cycles of 95 °C for 5 s, 58 °C for 20 s, and 72 °C for 30 s in a 96-well plate. An ABI 7500 real-time PCR system (Applied Biosystems, Foster City, CA, USA) was used to run all qRT-PCRs. Three biological replicates and three technical replicates were conducted for each sample. The relative expression levels of *GhCIPK* genes were calculated by the 2^-ΔΔ*C*t^ method [52].

The expression value (FPKMs, fragments per kilobase per million reads) of the *GhCIPK* gene family was extracted from the RNA-seq data in various *G. hirsutum* acc. TM-1 tissues (PRJNA248163) [34]. Gene expression levels were calculated as FPKM. MeV 4.0 software was used to generate heat maps of the *GhCIPK* genes.

### 4.7. Identification of Single Nucleotide Polymorphisms (SNPs) for CIPK Genes and Correlation Analysis with Cottonseed Oil and Protein Content Traits

To identify the single nucleotide polymorphism (SNPs) for *CIPK* genes, the RNA-seq data from CRI 36 and Hai 7124 was analyzed, and SNPs were scanned using SOAPsnp software [84]. CRI 36 is currently a commercial cultivar of *G. hirsutum* and Hai 7124 is a non-commercial *G. barbadense.* We used an interspecific backcross inbred line (BIL) population of 180 lines derived from CRI 36 and Hai 7124 to validate SNP markers of cotton *CIPKs*. SNP primers for *CIPK* genes were designed using Primer v5.0 software (Table S11), and high-resolution melting (HRM) was used to analyze the relationship of SNPs and cottonseed oil content as described in previous articles [48]. The simple correlation analysis was performed using SPPS software (IBM, New York, NY, USA).

### 4.8. Genetic Transformation, Oil Content Detection, and Fatty Acid Composition Analysis

Combined with RT-PCR and rapid amplification of cDNA ends technique (RACE), the full length of *GhCIPK6* was obtained from ‘ZG5′ cDNA. The plant binary vector pBI121, which carried the *NPT II* (neomycin phosphotransferase II) gene, was used for expression of the *GhCIPK6* gene under the control of the CaMV 35S promoter. The ‘11-0516′ upland cotton cultivar was grown in the field until flowering, and after self-pollination for 24 h, the plasmid of recombinant vector (pBI121-GhCIPK6) was injected into the ovary of ‘11-0516′ by syringe. These injected mature seeds were grown again, and positive transgenic plants were detected using the method described in previous reports [85,86]. According to the manufacturer’s instructions of DIG High Prime DNA Labeling and Detection Starter kit I (Roche, Basel, Switzerland), Southern blotting was conducted using the NPT II gene as the DNA probe to detect *GhCIPK6* copies. 

The seed oil contents of transgenic cotton plants and wild-type plants were detected in approximately 1 g of seeds of each sample by an NMI20-Analyst nuclear magnetic resonance spectrometer (Niumag, Shanghai, China). The fatty acid composition of each cotton sample was analyzed using a gas chromatograph (GC-2030, Shimadzu, Kyoto, Japan) equipped with a flame ionization detector (FID) and an SH-Rtx-65 capillary column (30 m × 0.25 mm × 0.50 μm). Samples (1 µL), including fatty acids and the n-hexane solution, were injected at 280 °C in split mode (30:1). All oil content and fatty acid composition data were analyzed by Student’s *t*-*test*. Differences were considered statistically significant when *p* < 0.05 (*) compared with the control.

## 5. Conclusions

In the present study, a total of 158 *CIPK* genes from *G. hirsutum* and *G. barbadense* were identified. Among them, phylogenetic classifications, gene structures, motifs, chromosomal localizations, and duplication genes were analyzed. Segmental duplication was a major impetus for the expansion of cotton *CIPK* gene family, and purifying selection played a major role in the evolution of the gene family. Additionally, *cis-*elements, expression patterns, and co-localization relationship of *GhCIPK* genes with oil or protein QTLs were also analyzed. Most of *GhCIPK* genes were not associated with natural variations in cotton oil content. Overexpression of *GhCIPK6* gene could reduce oil content and change fatty acid composition. Additionally, the relationships of miRNAs and their target *GhCIPK* genes showed that three genes were regulated by miRNAs. Our results provide a solid foundation for further studies of the roles of *CIPK* genes in stress responses and oil synthesis.

## Figures and Tables

**Table 1:** Ka/Ks analysis for the duplicated *CIPK* gene pairs in *G. hirsutum* and *G. barbadense*.

Species	Duplicated Gene 1	Duplicated Gene 2	Ka	Ks	Ka/Ks	Purifying Selection	Duplicate Type
*G. hirsutum*	GhCIPK1	GhCIPK38	0	0.0296	0	No	Segmental
	GhCIPK10	GhCIPK47	0.0081	0	0	No	Segmental
	GhCIPK10	GhCIPK30	0.0985	0.7646	0.1288	Yes	Segmental
	GhCIPK10	GhCIPK68	0.0985	0.7646	0.1288	Yes	Segmental
	GhCIPK10	GhCIPK55	0.0983	0.3954	0.2486	Yes	Segmental
	GhCIPK10	GhCIPK16	0.0983	0.3474	0.283	Yes	Segmental
	GhCIPK11	GhCIPK49	0.0165	0.0282	0.5851	Yes	Segmental
	GhCIPK11	GhCIPK72	0.1436	0.5354	0.2682	Yes	Segmental
	GhCIPK12	GhCIPK51	0	0.0619	0	No	Segmental
	GhCIPK13	GhCIPK20	0.0905	0.7899	0.1146	Yes	Segmental
	GhCIPK13	GhCIPK37	0.0959	0.7194	0.1333	Yes	Segmental
	GhCIPK13	GhCIPK43	0.0822	0.9098	0.0903	Yes	Segmental
	GhCIPK13	GhCIPK52	0	0	0	No	Segmental
	GhCIPK13	GhCIPK59	0.0997	0.7138	0.1397	Yes	Segmental
	GhCIPK13	GhCIPK74	0.1052	0.5893	0.1785	Yes	Segmental
	GhCIPK14	GhCIPK53	0.0242	0.031	0.7806	Yes	Segmental
	GhCIPK15	GhCIPK54	0.0163	0.0294	0.5544	Yes	Segmental
	GhCIPK16	GhCIPK30	0.089	0.5674	0.1569	Yes	Segmental
	GhCIPK16	GhCIPK55	0	0.0303	0	No	Segmental
	GhCIPK16	GhCIPK68	0.089	0.4512	0.1973	Yes	Segmental
	GhCIPK18	GhCIPK56	0.0082	0	0	No	Segmental
	GhCIPK19	GhCIPK25	0.092	0.4042	0.2276	Yes	Segmental
	GhCIPK19	GhCIPK57	0.016	0	0	No	Segmental
	GhCIPK19	GhCIPK64	0.101	0.4042	0.2499	Yes	Segmental
	GhCIPK2	GhCIPK39	0.008	0	0	No	Segmental
	GhCIPK20	GhCIPK59	0.0081	0.0606	0.1337	Yes	Segmental
	GhCIPK20	GhCIPK74	0.0765	0.7726	0.099	Yes	Segmental
	GhCIPK20	GhCIPK43	0.0812	0.5899	0.1377	Yes	Segmental
	GhCIPK20	GhCIPK52	0.0905	0.7899	0.1146	Yes	Segmental
	GhCIPK20	GhCIPK37	0.0676	0.8566	0.0789	Yes	Segmental
	GhCIPK21	GhCIPK44	0.1332	0.742	0.1795	Yes	Segmental
	GhCIPK21	GhCIPK53	0.1358	0.7711	0.1761	Yes	Segmental
	GhCIPK21	GhCIPK60	0	0.0289	0	No	Segmental
	GhCIPK22	GhCIPK28	0.0673	0.3641	0.1848	Yes	Segmental
	GhCIPK22	GhCIPK45	0.0495	0.4803	0.1031	Yes	Segmental
	GhCIPK22	GhCIPK61	0.0081	0.0296	0.2736	Yes	Segmental
	GhCIPK22	GhCIPK66	0.0585	0.4125	0.1418	Yes	Segmental
	GhCIPK23	GhCIPK35	0.1488	0.405	0.3674	Yes	Segmental
	GhCIPK23	GhCIPK62	0	0.0284	0	No	Segmental
	GhCIPK23	GhCIPK73	0.1488	0.3597	0.4137	Yes	Segmental
	GhCIPK24	GhCIPK63	0.0083	0.0541	0.1534	Yes	Segmental
	GhCIPK25	GhCIPK57	0.0927	0.3908	0.2372	Yes	Segmental
	GhCIPK25	GhCIPK64	0.024	0	0	No	Segmental
	GhCIPK26	GhCIPK80	0.0159	0.1762	0.0902	Yes	Segmental
	GhCIPK27	GhCIPK65	0	0.0586	0	No	Segmental
	GhCIPK28	GhCIPK45	0.058	0.611	0.0949	Yes	Segmental
	GhCIPK28	GhCIPK61	0.0757	0.422	0.1794	Yes	Segmental
	GhCIPK28	GhCIPK66	0.0081	0.0929	0.0872	Yes	Segmental
	GhCIPK29	GhCIPK67	0.0158	0	0	No	Segmental
	GhCIPK3	GhCIPK32	0.6002	3.0016	0.2	Yes	Segmental
	GhCIPK3	GhCIPK40	0.5373	0	0	No	Segmental
	GhCIPK3	GhCIPK71	0.6244	2.6547	0.2352	Yes	Segmental
	GhCIPK30	GhCIPK47	0.1077	0.7646	0.1409	Yes	Segmental
	GhCIPK30	GhCIPK55	0.089	0.633	0.1406	Yes	Segmental
	GhCIPK30	GhCIPK68	0	0.0609	0	No	Segmental
	GhCIPK31	GhCIPK69	0	0.0296	0	No	Segmental
	GhCIPK32	GhCIPK40	0.1707	0.3898	0.4379	Yes	Segmental
	GhCIPK32	GhCIPK71	0.0078	0	0	No	Segmental
	GhCIPK33	GhCIPK70	0.0081	0.0291	0.2784	Yes	Segmental
	GhCIPK34	GhCIPK72	0.0166	0.0564	0.2943	Yes	Segmental
	GhCIPK35	GhCIPK62	0.1488	0.4532	0.3283	Yes	Segmental
	GhCIPK35	GhCIPK73	0.0167	0.027	0.6185	Yes	Segmental
	GhCIPK37	GhCIPK43	0.0638	0.5677	0.1124	Yes	Segmental
	GhCIPK37	GhCIPK52	0.0959	0.7194	0.1333	Yes	Segmental
	GhCIPK37	GhCIPK59	0.0765	0.8566	0.0893	Yes	Segmental
	GhCIPK37	GhCIPK74	0.0082	0.0574	0.1429	Yes	Segmental
	GhCIPK4	GhCIPK42	0.0082	0	0	No	Segmental
	GhCIPK40	GhCIPK71	0.1604	0.3953	0.4058	Yes	Segmental
	GhCIPK43	GhCIPK52	0.0822	0.9098	0.0903	Yes	Segmental
	GhCIPK43	GhCIPK59	0.0902	0.4754	0.1897	Yes	Segmental
	GhCIPK43	GhCIPK74	0.0728	0.5677	0.1282	Yes	Segmental
	GhCIPK44	GhCIPK53	0.1271	0.6696	0.1898	Yes	Segmental
	GhCIPK44	GhCIPK60	0.1331	0.6735	0.1976	Yes	Segmental
	GhCIPK45	GhCIPK61	0.0409	0.5525	0.074	Yes	Segmental
	GhCIPK45	GhCIPK66	0.0494	0.6816	0.0725	Yes	Segmental
	GhCIPK47	GhCIPK68	0.1077	0.7646	0.1409	Yes	Segmental
	GhCIPK48	GhCIPK50	0.0418	0.1206	0.3466	Yes	Segmental
	GhCIPK48	GhCIPK75	0	0.0574	0	No	Segmental
	GhCIPK48	GhCIPK76	0.0418	0.189	0.2212	Yes	Segmental
	GhCIPK49	GhCIPK72	0.1336	0.4847	0.2756	Yes	Segmental
	GhCIPK5	GhCIPK41	0.0161	0.0305	0.5279	Yes	Segmental
	GhCIPK50	GhCIPK75	0.0418	0.1206	0.3466	Yes	Segmental
	GhCIPK50	GhCIPK76	0	0.0574	0	No	Segmental
	GhCIPK52	GhCIPK59	0.0997	0.7138	0.1397	Yes	Segmental
	GhCIPK52	GhCIPK68	0.3312	2.0206	0.1639	Yes	Segmental
	GhCIPK55	GhCIPK74	0.3463	1.4874	0.2328	Yes	Segmental
	GhCIPK57	GhCIPK64	0.1017	0.3908	0.2602	Yes	Segmental
	GhCIPK58	GhCIPK77	0.0246	0.0908	0.2709	Yes	Segmental
	GhCIPK59	GhCIPK74	0.0855	0.7726	0.1107	Yes	Segmental
	GhCIPK6	GhCIPK13	0.0822	0.9098	0.0903	Yes	Segmental
	GhCIPK6	GhCIPK20	0.0812	0.5899	0.1377	Yes	Segmental
	GhCIPK6	GhCIPK37	0.0638	0.5677	0.1124	Yes	Segmental
	GhCIPK6	GhCIPK43	0	0	0	No	Segmental
	GhCIPK6	GhCIPK52	0.0822	0.9098	0.0903	Yes	Segmental
	GhCIPK6	GhCIPK59	0.0902	0.4754	0.1897	Yes	Segmental
	GhCIPK6	GhCIPK74	0.0728	0.5677	0.1282	Yes	Segmental
	GhCIPK61	GhCIPK66	0.0669	0.4754	0.1407	Yes	Segmental
	GhCIPK62	GhCIPK73	0.1488	0.405	0.3674	Yes	Segmental
	GhCIPK7	GhCIPK21	0.1424	0.6837	0.2083	Yes	Segmental
	GhCIPK7	GhCIPK44	0.0082	0.028	0.2929	Yes	Segmental
	GhCIPK7	GhCIPK60	0.1423	0.76	0.1872	Yes	Segmental
	GhCIPK75	GhCIPK76	0.0418	0.154	0.2714	Yes	Segmental
	GhCIPK78	GhCIPK79	0	0	0	No	Segmental
	GhCIPK8	GhCIPK22	0.0495	0.4803	0.1031	Yes	Segmental
	GhCIPK8	GhCIPK28	0.058	0.611	0.0949	Yes	Segmental
	GhCIPK8	GhCIPK45	0	0	0	No	Segmental
	GhCIPK8	GhCIPK61	0.0409	0.5525	0.074	Yes	Segmental
	GhCIPK8	GhCIPK66	0.0494	0.6816	0.0725	Yes	Segmental
	GhCIPK9	GhCIPK46	0.0329	0.0293	1.1229	No	Segmental
*G. barbadense*	GbCIPK1	GbCIPK37	0.0113	0.0325	0.3477	Yes	Segmental
	GbCIPK2	GbCIPK38	0.0228	0.0581	0.3924	Yes	Segmental
	GbCIPK4	GbCIPK41	0.0069	0.0391	0.1765	Yes	Segmental
	GbCIPK5	GbCIPK40	0.0113	0.0165	0.6848	Yes	Segmental
	GbCIPK6	GbCIPK14	0.13	0.4949	0.2627	Yes	Segmental
	GbCIPK6	GbCIPK36	0.104	0.435	0.2391	Yes	Segmental
	GbCIPK6	GbCIPK42	0.0068	0.0241	0.2822	Yes	Segmental
	GbCIPK6	GbCIPK51	0.13	0.5103	0.2548	Yes	Segmental
	GbCIPK6	GbCIPK59	0.0948	0.571	0.166	Yes	Segmental
	GbCIPK6	GbCIPK76	0.1015	0.4327	0.2346	Yes	Segmental
	GbCIPK6	GbCIPK78	0.0974	0.5699	0.1709	Yes	Segmental
	GbCIPK7	GbCIPK21	0.118	0.3925	0.3006	Yes	Segmental
	GbCIPK7	GbCIPK43	0.0134	0.0258	0.5194	Yes	Segmental
	GbCIPK7	GbCIPK60	0.1231	0.3939	0.3125	Yes	Segmental
	GbCIPK8	GbCIPK22	0.0663	0.2358	0.2812	Yes	Segmental
	GbCIPK8	GbCIPK44	0.0114	0.0078	1.4615	No	Segmental
	GbCIPK8	GbCIPK61	0.0665	0.2545	0.2613	Yes	Segmental
	GbCIPK8	GbCIPK67	0.0904	0.378	0.2392	Yes	Segmental
	GbCIPK9	GbCIPK45	0.0113	0.0588	0.1922	Yes	Segmental
	GbCIPK10	GbCIPK16	0.1053	0.3484	0.3022	Yes	Segmental
	GbCIPK10	GbCIPK30	0.1323	0.477	0.2774	Yes	Segmental
	GbCIPK10	GbCIPK46	0.009	0.0083	1.0843	No	Segmental
	GbCIPK10	GbCIPK70	0.1322	0.4774	0.2769	Yes	Segmental
	GbCIPK11	GbCIPK47	0.0023	0.0241	0.0954	Yes	Segmental
	GbCIPK11	GbCIPK49	0.0442	0.2282	0.1937	Yes	Segmental
	GbCIPK11	GbCIPK77	0.0466	0.2171	0.2146	Yes	Segmental
	GbCIPK12	GbCIPK34	0.0948	0.3398	0.279	Yes	Segmental
	GbCIPK12	GbCIPK48	0.0068	0.0161	0.4224	Yes	Segmental
	GbCIPK12	GbCIPK74	0.0922	0.3275	0.2815	Yes	Segmental
	GbCIPK13	GbCIPK50	0.0045	0	0	No	Segmental
	GbCIPK14	GbCIPK36	0.1155	0.4284	0.2696	Yes	Segmental
	GbCIPK14	GbCIPK42	0.1325	0.4982	0.266	Yes	Segmental
	GbCIPK14	GbCIPK51	0	0.0158	0	No	Segmental
	GbCIPK14	GbCIPK59	0.1209	0.5682	0.2128	Yes	Segmental
	GbCIPK14	GbCIPK76	0.1103	0.4125	0.2674	Yes	Segmental
	GbCIPK14	GbCIPK78	0.1263	0.5671	0.2227	Yes	Segmental
	GbCIPK15	GbCIPK52	0.0067	0.0418	0.1603	Yes	Segmental
	GbCIPK16	GbCIPK30	0.0985	0.5022	0.1961	Yes	Segmental
	GbCIPK16	GbCIPK54	0.0067	0.0248	0.2702	Yes	Segmental
	GbCIPK16	GbCIPK70	0.0933	0.5184	0.18	Yes	Segmental
	GbCIPK17	GbCIPK27	0.0799	0.2076	0.3849	Yes	Segmental
	GbCIPK17	GbCIPK55	0.0022	0.0257	0.0856	Yes	Segmental
	GbCIPK17	GbCIPK66	0.0762	0.2021	0.377	Yes	Segmental
	GbCIPK18	GbCIPK56	0.0022	0.1048	0.021	Yes	Segmental
	GbCIPK19	GbCIPK57	0.0114	0.0656	0.1738	Yes	Segmental
	GbCIPK20	GbCIPK58	0.0249	0.0339	0.7345	Yes	Segmental
	GbCIPK21	GbCIPK43	0.1207	0.404	0.2988	Yes	Segmental
	GbCIPK21	GbCIPK52	0.1214	0.4081	0.2975	Yes	Segmental
	GbCIPK21	GbCIPK60	0.009	0.0163	0.5521	Yes	Segmental
	GbCIPK22	GbCIPK44	0.4258	1.6158	0.2635	Yes	Segmental
	GbCIPK22	GbCIPK61	0.0045	0.0317	0.142	Yes	Segmental
	GbCIPK22	GbCIPK67	0.0953	0.3812	0.25	Yes	Segmental
	GbCIPK23	GbCIPK35	0.0848	0.3013	0.2814	Yes	Segmental
	GbCIPK23	GbCIPK62	0.0045	0.0082	0.5488	Yes	Segmental
	GbCIPK23	GbCIPK75	0.0823	0.3137	0.2624	Yes	Segmental
	GbCIPK24	GbCIPK63	0.009	0.0586	0.1536	Yes	Segmental
	GbCIPK25	GbCIPK57	0.0812	0.3393	0.2393	Yes	Segmental
	GbCIPK25	GbCIPK64	0.0068	0.0078	0.8718	Yes	Segmental
	GbCIPK26	GbCIPK65	0.025	0.0593	0.4216	Yes	Segmental
	GbCIPK27	GbCIPK55	0.0811	0.1914	0.4237	Yes	Segmental
	GbCIPK27	GbCIPK66	0.0022	0.0081	0.2716	Yes	Segmental
	GbCIPK27	GbCIPK71	0.1188	0.2344	0.5068	Yes	Segmental
	GbCIPK28	GbCIPK68	0.0046	0.0076	0.6053	Yes	Segmental
	GbCIPK29	GbCIPK69	0.0228	0.0245	0.9306	Yes	Segmental
	GbCIPK3	GbCIPK32	0.1067	0.433	0.2464	Yes	Segmental
	GbCIPK3	GbCIPK39	0.0136	0.0409	0.3325	Yes	Segmental
	GbCIPK3	GbCIPK73	0.099	0.4178	0.237	Yes	Segmental
	GbCIPK30	GbCIPK46	0.1268	0.4942	0.2566	Yes	Segmental
	GbCIPK30	GbCIPK54	0.1062	0.5344	0.1987	Yes	Segmental
	GbCIPK30	GbCIPK70	0.0068	0.0238	0.2857	Yes	Segmental
	GbCIPK31	GbCIPK71	0.0045	0.0081	0.5556	Yes	Segmental
	GbCIPK32	GbCIPK72	0.4802	1.8172	0.2643	Yes	Segmental
	GbCIPK33	GbCIPK73	0.0114	0.0315	0.3619	Yes	Segmental
	GbCIPK34	GbCIPK48	0.0998	0.3671	0.2719	Yes	Segmental
	GbCIPK34	GbCIPK74	0.0091	0.0406	0.2241	Yes	Segmental
	GbCIPK35	GbCIPK62	0.0848	0.3013	0.2814	Yes	Segmental
	GbCIPK35	GbCIPK75	0.0067	0.0257	0.2607	Yes	Segmental
	GbCIPK36	GbCIPK42	0.1065	0.4237	0.2514	Yes	Segmental
	GbCIPK36	GbCIPK51	0.1155	0.4284	0.2696	Yes	Segmental
	GbCIPK36	GbCIPK59	0.1143	0.4103	0.2786	Yes	Segmental
	GbCIPK36	GbCIPK76	0.0183	0.0158	1.1582	No	Segmental
	GbCIPK36	GbCIPK78	0.1222	0.4095	0.2984	Yes	Segmental
	GbCIPK39	GbCIPK73	0.1041	0.4619	0.2254	Yes	Segmental
	GbCIPK42	GbCIPK51	0.4678	1.2446	0.3759	Yes	Segmental
	GbCIPK42	GbCIPK59	0.4547	1.5066	0.3018	Yes	Segmental
	GbCIPK42	GbCIPK76	0.4425	1.2039	0.3676	Yes	Segmental
	GbCIPK42	GbCIPK78	0.4591	1.5003	0.306	Yes	Segmental
	GbCIPK43	GbCIPK60	0.1206	0.4054	0.2975	Yes	Segmental
	GbCIPK44	GbCIPK61	0.0639	0.2674	0.239	Yes	Segmental
	GbCIPK44	GbCIPK67	0.0928	0.3942	0.2354	Yes	Segmental
	GbCIPK46	GbCIPK70	0.1268	0.4946	0.2564	Yes	Segmental
	GbCIPK47	GbCIPK49	0.0418	0.1971	0.2121	Yes	Segmental
	GbCIPK47	GbCIPK77	0.0442	0.1865	0.237	Yes	Segmental
	GbCIPK48	GbCIPK74	0.0972	0.3542	0.2744	Yes	Segmental
	GbCIPK49	GbCIPK77	0.0045	0.0158	0.2848	Yes	Segmental
	GbCIPK51	GbCIPK59	0.1209	0.5516	0.2192	Yes	Segmental
	GbCIPK51	GbCIPK76	0.1103	0.4125	0.2674	Yes	Segmental
	GbCIPK51	GbCIPK78	0.1263	0.5505	0.2294	Yes	Segmental
	GbCIPK54	GbCIPK70	0.101	0.5514	0.1832	Yes	Segmental
	GbCIPK55	GbCIPK66	0.0786	0.1809	0.4345	Yes	Segmental
	GbCIPK57	GbCIPK64	0.0761	0.354	0.215	Yes	Segmental
	GbCIPK59	GbCIPK78	0.0113	0	0	No	Segmental
	GbCIPK59	GbCIPK76	0.1092	0.4081	0.2676	Yes	Segmental
	GbCIPK61	GbCIPK67	0.0905	0.4023	0.225	Yes	Segmental
	GbCIPK62	GbCIPK75	0.0823	0.3137	0.2624	Yes	Segmental
	GbCIPK66	GbCIPK71	0.1162	0.2235	0.5199	Yes	Segmental
	GbCIPK76	GbCIPK78	0.1171	0.4074	0.2874	Yes	Segmental

## Supplementary Materials

Supplementary materials can be found at https://www.mdpi.com/1422-0067/21/3/863/s1. Table S1. The *CIPK* genes used to construct phylogenetic tree in other species. Table S2. The list of the *CIPK* genes identified in *G. hirsutum*. Table S3. The list of the *CIPK* genes identified in *G. barbadense*. Table S4. Detailed information about the protein kinase domain and the NAF domain in each cotton CIPK protein. Table S5. Orthologous *CIPK* genes in and between the four cotton species. Table S6. Homoeologs for *CIPK* genes in the two allotetraploid cotton species. Table S7. Duplication of *CIPK* genes in *G. hirsutum* and *G. barbadense*. Table S8. The list of *cis-*elements in the 1.5 kb promoter regions of *GhCIPKs*. Table S9. The list of TFs in the 1.5 kb promoter regions of *GhCIPKs*. Table S10. The information of 17 non-synonymous SNPs in *G. hirsutum* and *G. barbadense*. Table S11. The list of primers in our study. Table S12. Association analysis between SNP markers and cottonseed oil content in a BIL population. Table S13. The predicted details of miRNA-mediated targeting regulatory relations with *GhCIPK* gene family in cotton. Figure S1. The distribution of orthologous *CIPK* gene pairs in and between *G. raimondii*, *G. arboreum*, *G. hirsutum*, and *G. barbadense*. Figure S2. The distribution of homoeologs for *CIPK* gene pairs between *G. hirsutum* and *G. barbadense.* Figure S3. Phylogenetic relationship of *CIPK* genes from *G. raimondii*, *G. arboretum*, and *G. hirsutum*. Figure S4. Phylogenetic relationship of *CIPK* genes from *G. raimondii*, *G. arboretum*, and *G. barbadense*. Figure S5. Conserved motifs in the CIPK proteins in *G. hirsutum* and *G. barbadense*. The MEME program was used on all motifs of CIPKs from *G. hirsutum* and *G. barbadense*. Each colored box represents a motif in cotton CIPK proteins. Figure S6. Sequence logos of cotton CIPK proteins. Figure S7. The NAF domain in the CIPK proteins. The four cotton CIPK proteins were compared using the DNAMAN 7.0 software and conserved amino acid residues are shown in dark blue. Figure S8. A co-localization analysis of *GhCIPK* genes with cotton oil and protein content QTLs. *GhCIPK* genes are shown in red and underlining indicates the GhCIPK genes colocalized with oil or protein content QTLs. QTLs from the CottonQTL database [45,46] are indicated by black color, and QTLs from the recently published article [47] are indicated by red color. Figure S9. Characterization of alternative splicing (AS) events and validation of full-length isoforms using RT-PCR. (**A**) Classification of AS events; (**B**) RT-PCR validation of AS events for *GhCIPK17*. Figure S10. The relative expression levels of miRNAs and their putative targeting *GhCIPKs* in cotton ovules. Figure S11. Potential alternative spliced isoform structure of *GhCIPK48* and *GhCIPK51* targeted by cotton miRNAs. The dark red and red dotted rectangles were the predicted miRNA targeting sites. Exons are represented by green boxes and introns by dark lines.
