# Supplementary material for: Genome-Wide Characterization and Analysis of CIPK Gene Family in Two Cultivated Allopolyploid Cotton Species: Sequence Variation, Association with Seed Oil Content, and the Role of GhCIPK6
Source: Int J Mol Sci. 2020 Jan 29;21(3):863. doi: 10.3390/ijms21030863 (PMC7037685; doi:10.3390/ijms21030863)

Figure S7. The NAF domain in the CIPK proteins. The four cotton CIPK proteins were compared using the DNAMAN 7.0 software and conserved amino acid residues are shown in dark blue.

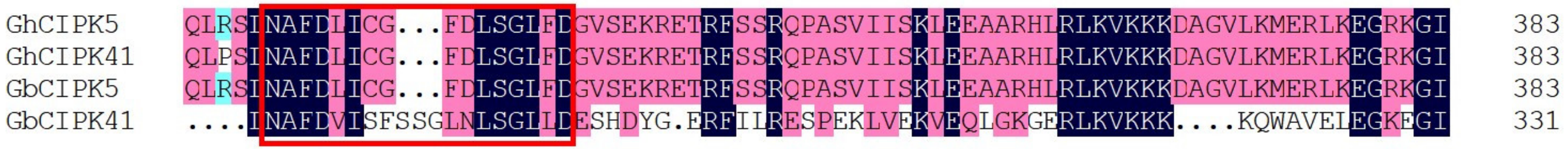

Supplement: Supplementary file 1 [file ijms-21-00863-s001.zip › ijms-686279-final/ijms-686279-supplementary/Figure S7.pdf]
