# Supplementary figures and images for: Genome-Wide Characterization and Analysis of CIPK Gene Family in Two Cultivated Allopolyploid Cotton Species: Sequence Variation, Association with Seed Oil Content, and the Role of GhCIPK6
Source: Int J Mol Sci. 2020 Jan 29;21(3):863. doi: 10.3390/ijms21030863 (PMC7037685; doi:10.3390/ijms21030863)

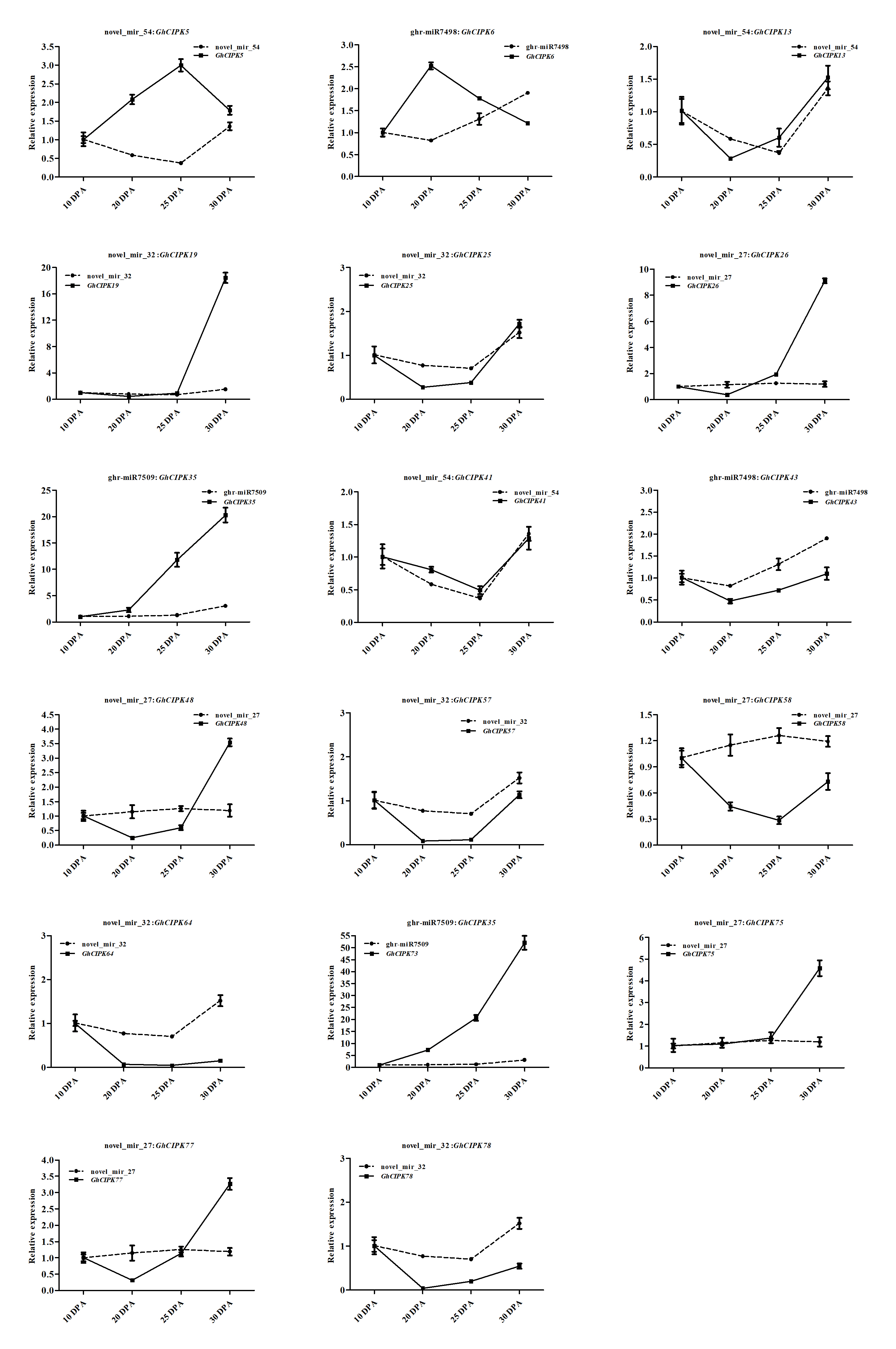

Supplement: Supplementary file 1 [file ijms-21-00863-s001.zip › ijms-686279-final/ijms-686279-supplementary/Figure S10.jpg]

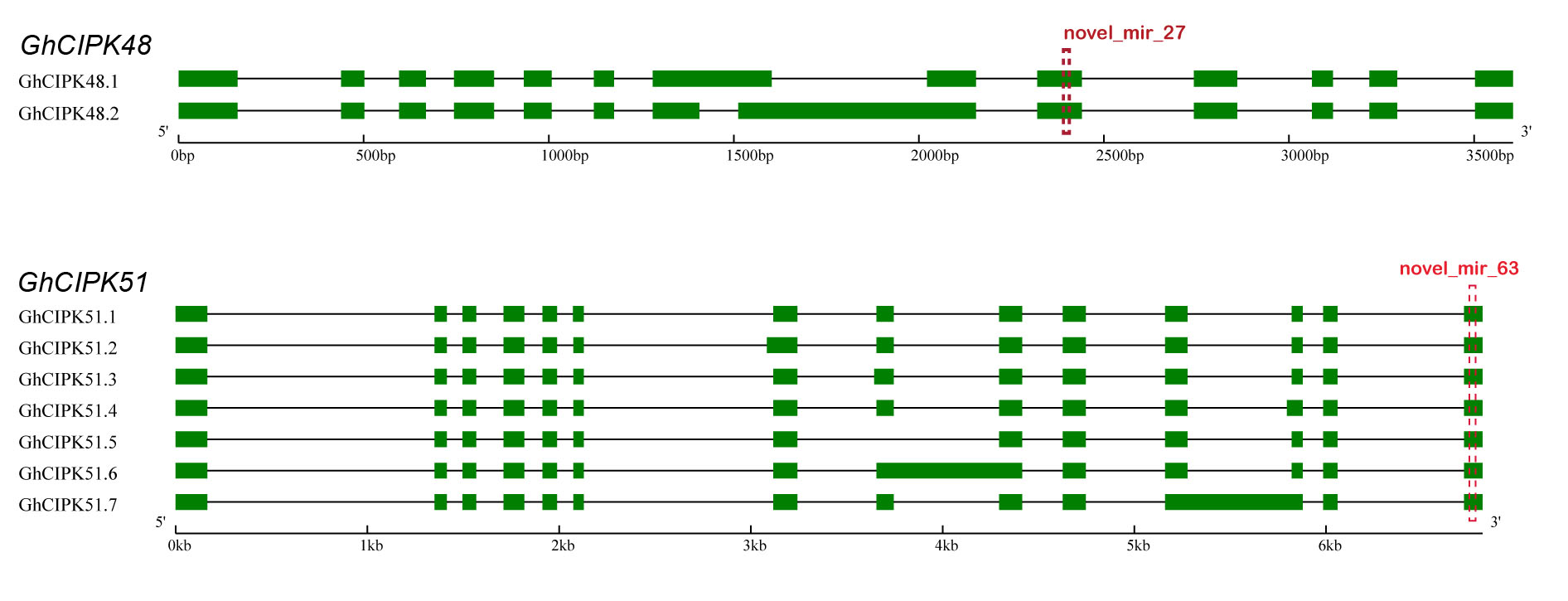

Supplement: Supplementary file 1 [file ijms-21-00863-s001.zip › ijms-686279-final/ijms-686279-supplementary/Figure S11.jpg]

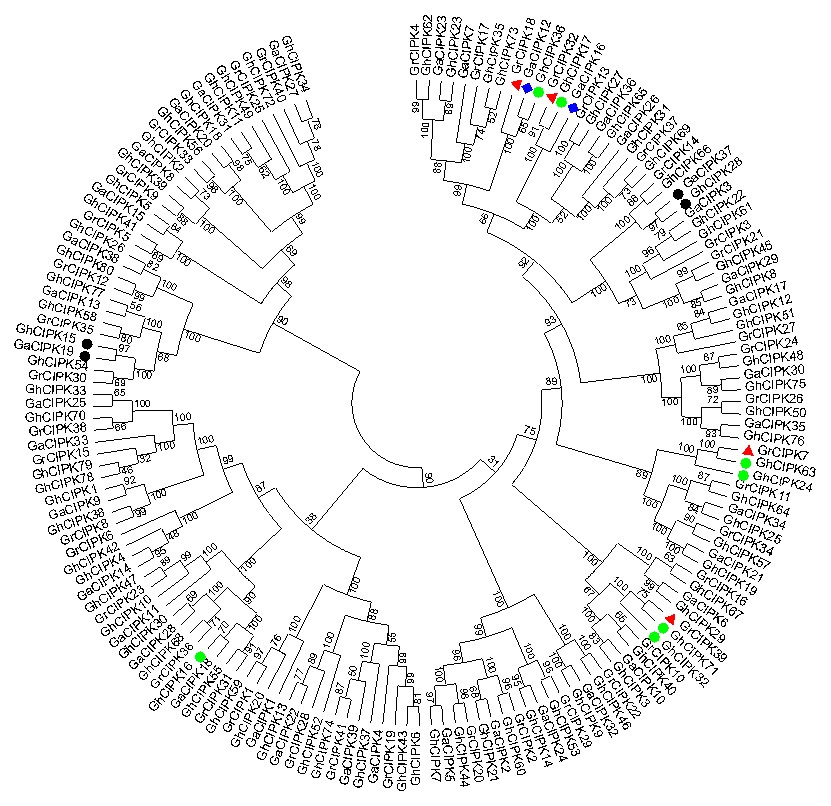

Supplement: Supplementary file 1 [file ijms-21-00863-s001.zip › ijms-686279-final/ijms-686279-supplementary/Figure S3.jpg]

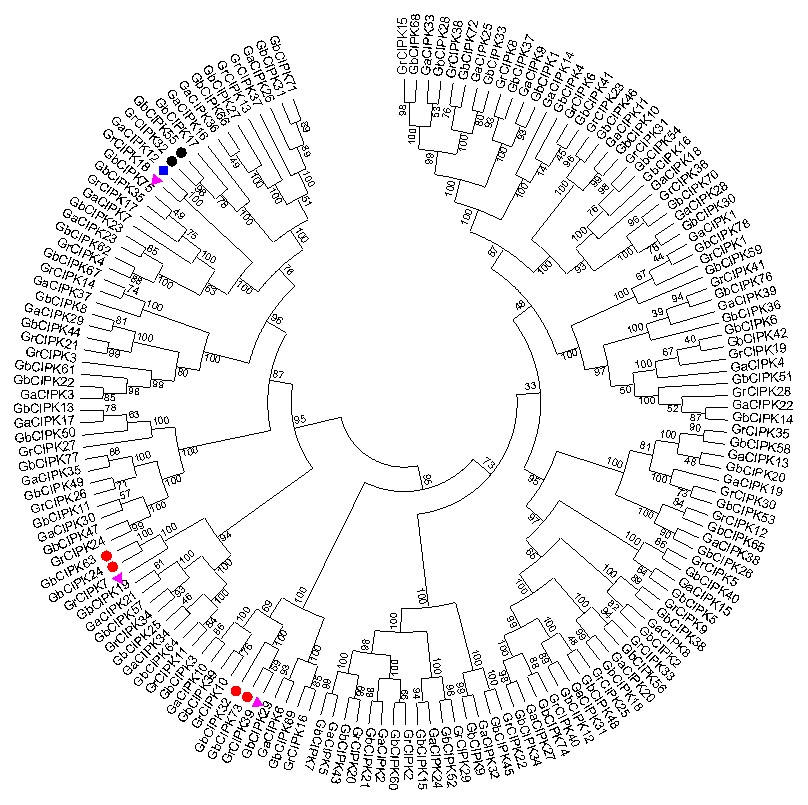

Supplement: Supplementary file 1 [file ijms-21-00863-s001.zip › ijms-686279-final/ijms-686279-supplementary/Figure S4.jpg]

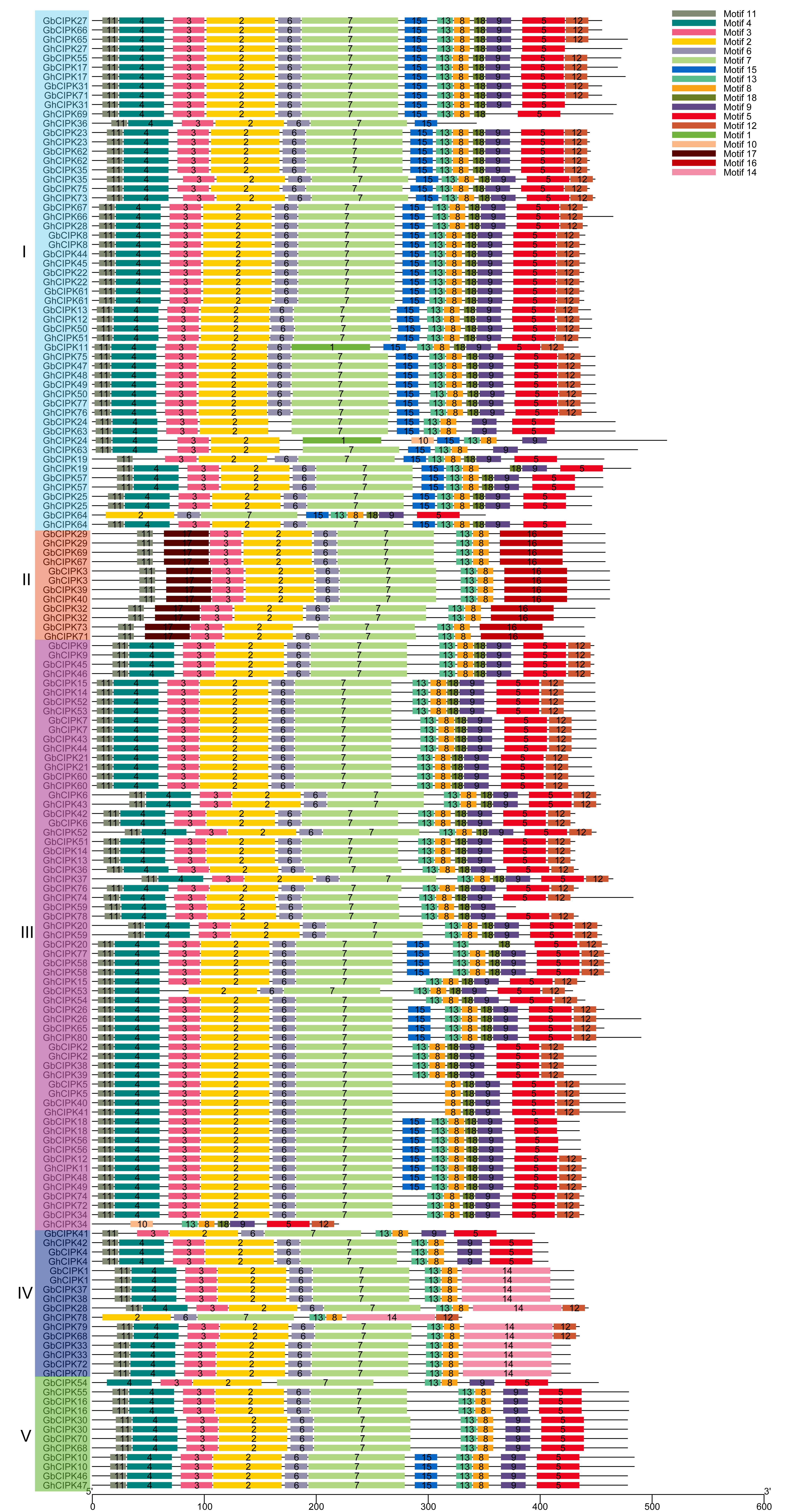

Supplement: Supplementary file 1 [file ijms-21-00863-s001.zip › ijms-686279-final/ijms-686279-supplementary/Figure S5.jpg]

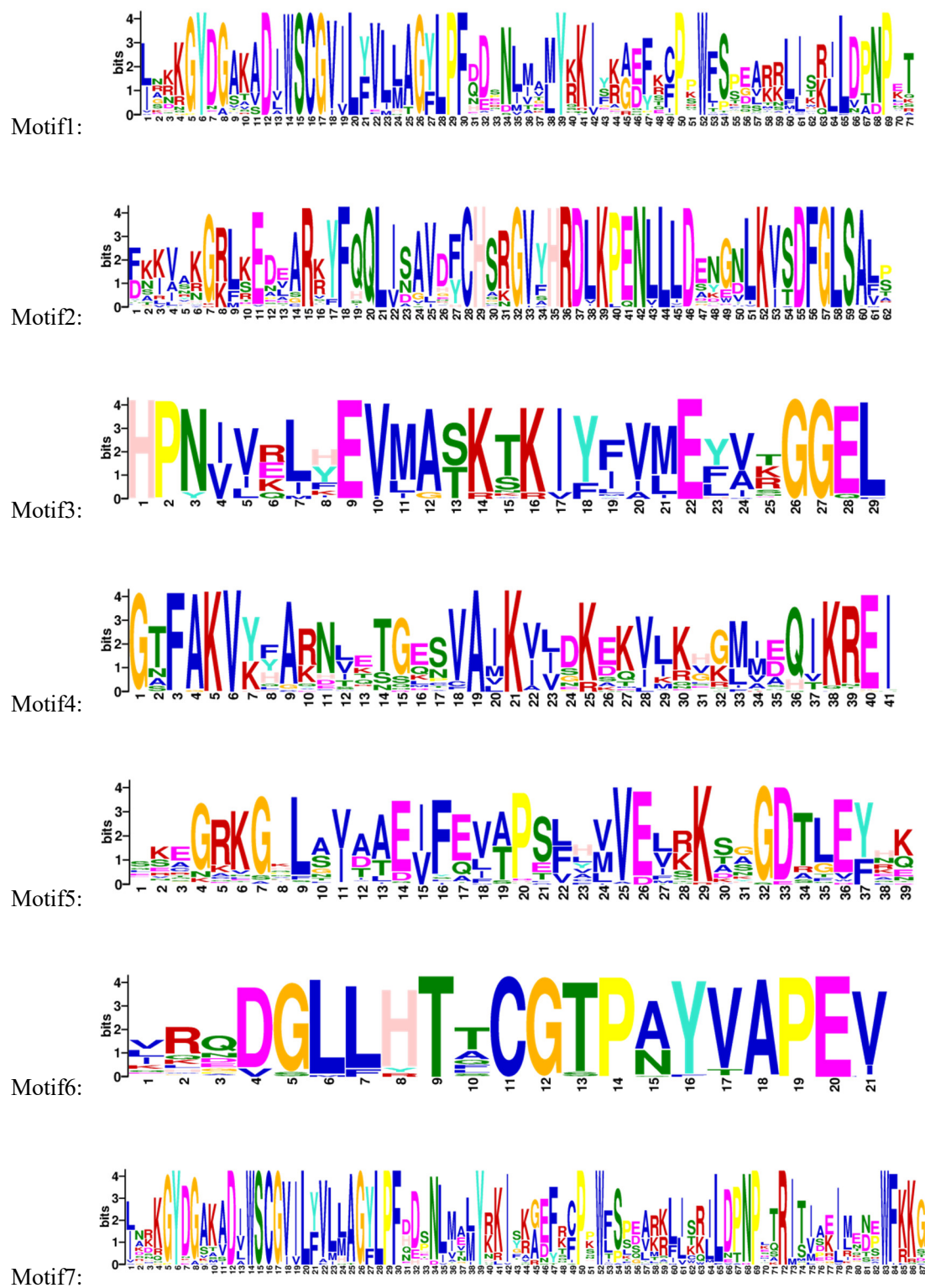

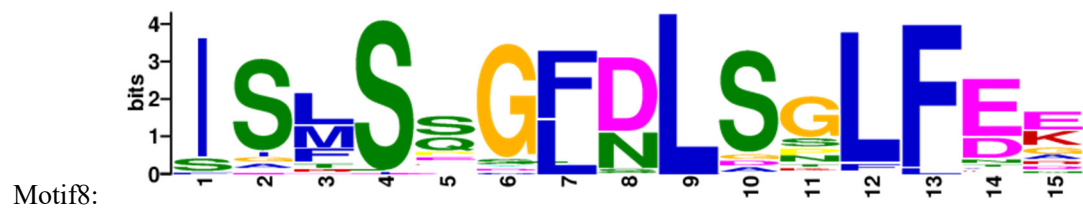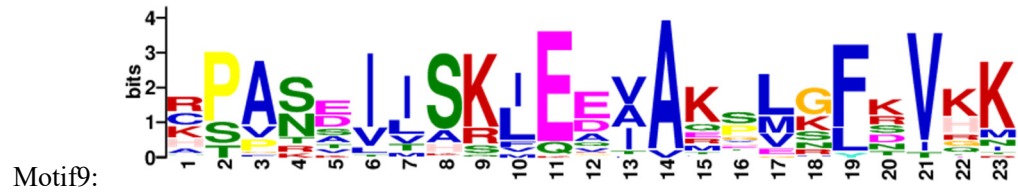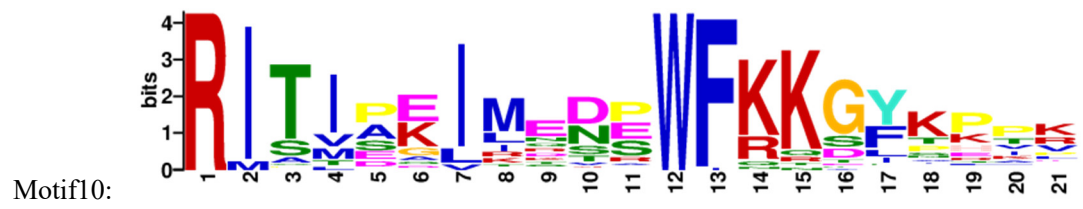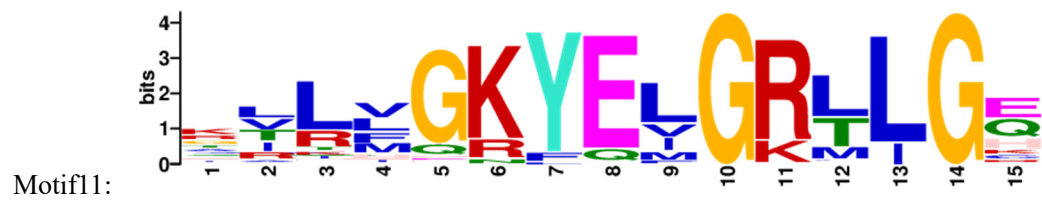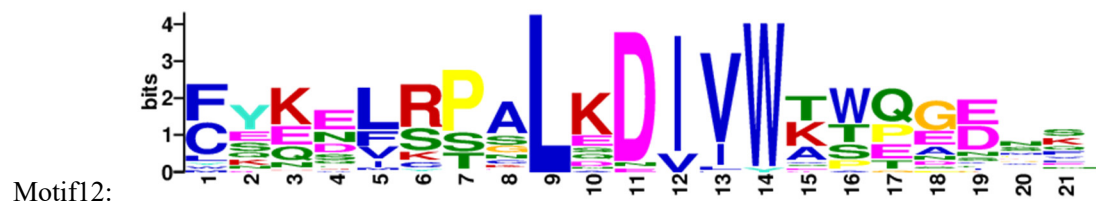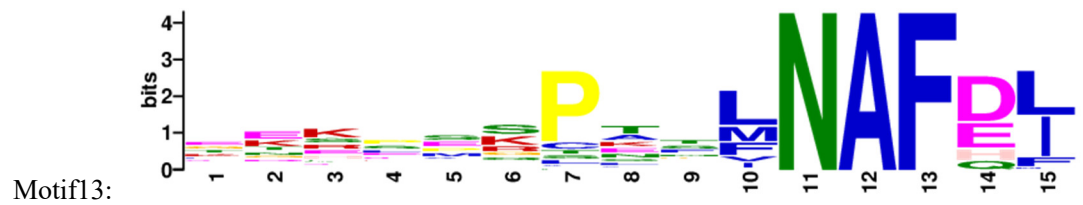

Motif14:

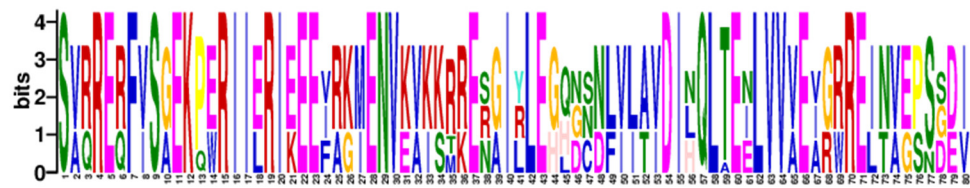

Motif15:

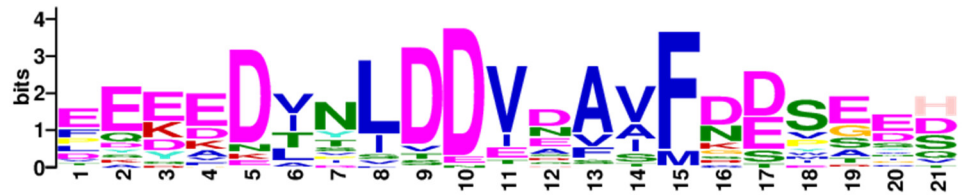

Motif16:

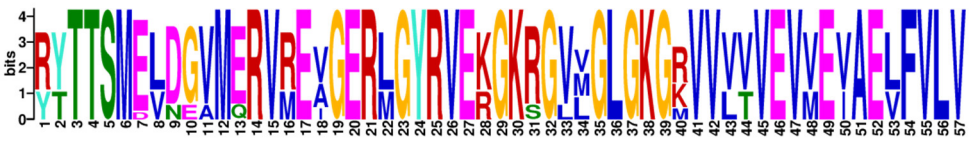

Motif17:

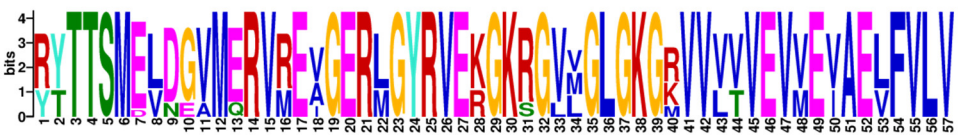

Motif18:

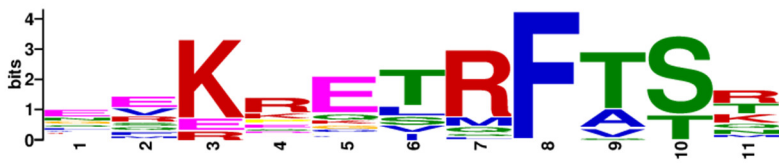

Supplement: Supplementary file 1 [file ijms-21-00863-s001.zip › ijms-686279-final/ijms-686279-supplementary/Figure S6.pdf]

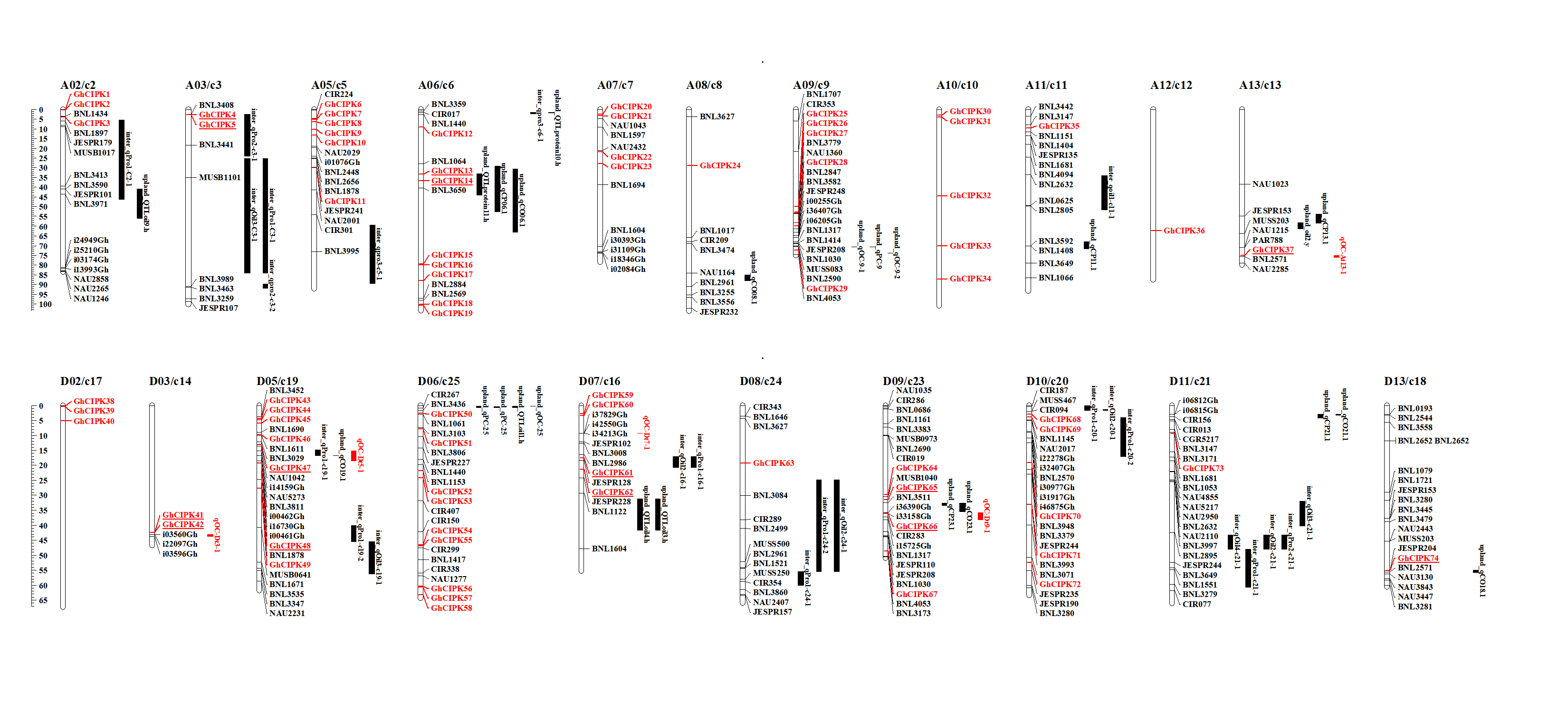

Supplement: Supplementary file 1 [file ijms-21-00863-s001.zip › ijms-686279-final/ijms-686279-supplementary/Figure S8.tif]

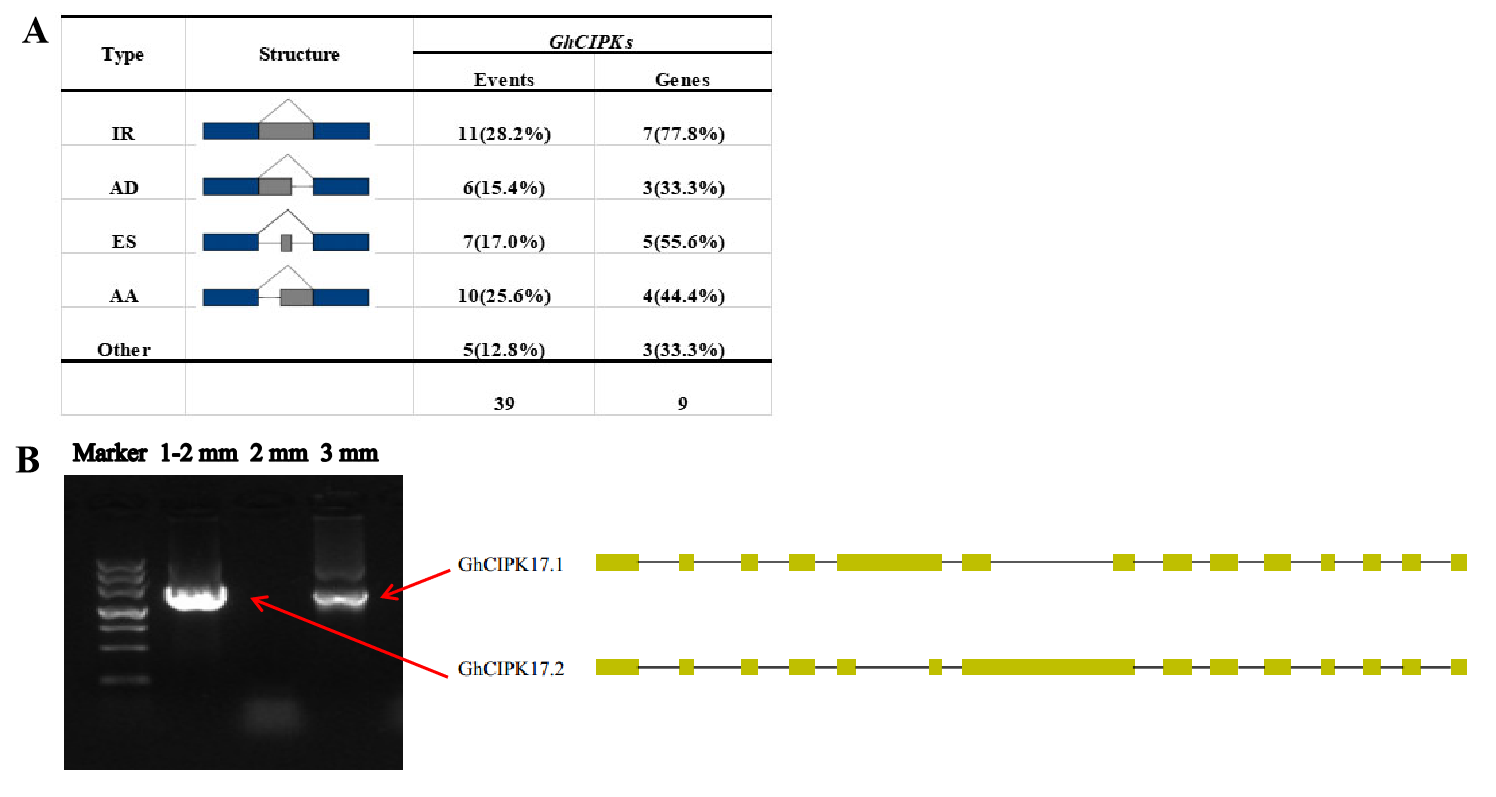

Supplement: Supplementary file 1 [file ijms-21-00863-s001.zip › ijms-686279-final/ijms-686279-supplementary/Figure S9.tif]
